# A Multiplex Protein Panel Applied to Cerebrospinal Fluid Reveals Three New Biomarker Candidates in ALS but None in Neuropathic Pain Patients

**DOI:** 10.1371/journal.pone.0149821

**Published:** 2016-02-25

**Authors:** Anne-Li Lind, Di Wu, Eva Freyhult, Constantin Bodolea, Titti Ekegren, Anders Larsson, Mats G. Gustafsson, Lenka Katila, Jonas Bergquist, Torsten Gordh, Ulf Landegren, Masood Kamali-Moghaddam

**Affiliations:** 1 Department of Surgical Sciences, Anaesthesiology and Intensive Care and Uppsala Berzelii Technology Center for Neurodiagnostics, Uppsala University, Uppsala, Sweden; 2 Department of Immunology, Genetics and Pathology, Science for Life Laboratory, Uppsala University, Uppsala, Sweden; 3 Department of Medical Sciences, Cancer Pharmacology and Computational Medicine, Science for Life Laboratory, Bioinformatics Infrastructure for Life Sciences, Uppsala University, Uppsala, Sweden; 4 Department of Anaesthesia and Intensive Care, University of Medicine and Pharmacy, Cluj, Napoca, Romania; 5 Department of Engineering Sciences, Uppsala University, Uppsala, Sweden; 6 Department of Medical Sciences, Biochemical Structure and Function, Uppsala University, Uppsala, Sweden; 7 Department of Medical Sciences, Cancer Pharmacology and Computational Medicine, Uppsala University, Uppsala, Sweden; 8 Department of Chemistry-BMC, Analytical Chemistry and Science for Life Laboratory, Uppsala University, Uppsala, Sweden; Children's Hospital of Pittsburgh, University of Pittsburgh Medical Center, UNITED STATES

## Abstract

The objective of this study was to develop and apply a novel multiplex panel of solid-phase proximity ligation assays (SP-PLA) requiring only 20 μL of samples, as a tool for discovering protein biomarkers for neurological disease and treatment thereof in cerebrospinal fluid (CSF). We applied the SP-PLA to samples from two sets of patients with poorly understood nervous system pathologies amyotrophic lateral sclerosis (ALS) and neuropathic pain, where patients were treated with spinal cord stimulation (SCS). Forty-seven inflammatory and neurotrophic proteins were measured in samples from 20 ALS patients and 15 neuropathic pain patients, and compared to normal concentrations in CSF from control individuals. Nineteen of the 47 proteins were detectable in more than 95% of the 72 controls. None of the 21 proteins detectable in CSF from neuropathic pain patients were significantly altered by SCS. The levels of the three proteins, follistatin, interleukin-1 alpha, and kallikrein-5 were all significantly reduced in the ALS group compared to age-matched controls. These results demonstrate the utility of purpose designed multiplex SP-PLA panels in CSF biomarker research for understanding neuropathological and neurotherapeutic mechanisms. The protein changes found in the CSF of ALS patients may be of diagnostic interest.

## Introduction

Many neurological conditions would benefit from molecular biomarkers for improved understanding of diseases and their susceptibility to treatment. Measurements of proteins in cerebrospinal fluid (CSF) provide valuable insights into the state of the central nervous system during disease and treatment [1]. However, adequate tools for parallel and sensitive protein analysis and biomarker discovery have been lacking. Optimal methods for analysis of precious CSF samples should provide high sensitivity, target selectivity, detection of multiple targets in parallel, and be sample sparing. The solid phase proximity ligation assay (SP-PLA) [2] and other high-performance DNA-assisted proximity assays [3, 4], meet these requirements [5–7] by using pairs or trios of antibodies with attached oligonucleotides. These technologies have identified biomarker candidates for cardiovascular diseases [6] and cancers [4, 8] using only 1–5 μL samples of blood plasma. Here we apply the technology for neurological investigations via CSF. We designed a novel multiplex SP-PLA (S1 Fig) protein panel for application in neurological research and tested its validity by applying it to two neuropathological and -therapeutic research questions as a proof of principle. Although each neurological disease and treatment can ultimately be expected to have a unique profile/fingerprint, several proteins appear on the “biomarker candidate hot list” of many neurological diseases. For example, a panel that includes inflammatory, glial, and neurotrophic proteins may be relevant for discovery stage investigations in several, if not most, diseases and treatments of the nervous system.

Amyotrophic lateral sclerosis (ALS) and neuropathic pain both lack established human CSF biomarker profiles. The molecular underpinnings of the devastating disease ALS remain essentially unknown despite extensive research efforts. Neuropathic pain, which affects 7–10% of the general population [9], is usually resistant to treatment but spinal cord stimulation (SCS)–with an unclear mechanism of action–tends to offer significant and prolonged pain relief for 60–70% of eligible neuropathic pain patients [10–13] where standard treatments fail. In spite of the differences, ample evidence indicates that inflammation, glial activation and neurotrophic support may have central roles in the pathophysiological processes of both ALS [14–24] and chronic pain [25–38]. We hypothesized that ALS as well as SCS-treatment of chronic pain, while representing distinct neurological conditions, might nonetheless both alter concentrations of CSF proteins. We therefore developed a multiplex panel of assays for 47 proteins–cytokines, glial markers, inflammatory mediators and neurotrophins human proteins (S1 Appendix). We applied this panel to CSF from ALS patients and neuropathic pain patients treated with SCS, as well as to neurologically normal controls.

## Materials and Methods

### Subjects

CSF was collected from 92 individuals without known neurological disorders undergoing spinal anesthesia for planned minor urology surgeries. Of these 92 controls, 20 were selected to match the age and sex of 20 patients with ALS. The remaining 72 (mean age 59.7 (21–81), 68 males) were used to obtain concentration values of CSF protein ranges in neurologically healthy individuals.

Twenty patients with confirmed ALS (mean age 64.9 (47–79), 8 males), and 20 age- and sex-matched control samples (mean age 66.1 (51–84), 10 male) were selected (S1 Table). The most common diagnosis was ALS with limb onset (n = 18), followed by ALS with bulbar onset (n = 2).

Fifteen patients (mean age 56.9 (47–68), 4 males) with long lasting neuropathic pain (median 10 years, range 3–23 years), and permanently implanted SCS since more than three months (median 3 years, range 1–10 years) with self-reported good pain relief, were included in the study. Pain diagnoses were radiculitis (n = 11), post-surgical pain (n = 1), phantom limb pain (n = 1), and polyneuropathy (n = 1). Neuropathic pain patients underwent two consecutive lumbar punctures, resulting in 30 paired CSF samples. Before the first lumbar puncture their stimulator was turned off for 48 hours (h) (except for one patient who chose 24h), keeping medications constant. After three weeks of normal SCS use they returned for the second lumbar puncture.

### Standard protocol approvals, registrations, and patient consents

The study was conducted in accordance with the Declaration of Helsinki. CSF samples from 127 individuals were included in this study, which was approved by the Regional Ethical Review Boards of Uppsala, Sweden and Cluj, Romania respectively, and undertaken with the written consent of the individual donors.

### CSF sample collection

All patient samples were collected using the same protocol. Briefly, fasting patients underwent lumbar puncture. After removal of the first sample aliquots to avoid blood contamination of the CSF from potential puncture bleeding, the samples were collected in polypropylene tubes, which were sealed, gently mixed and put on ice. The samples were centrifuged at 1,300 g for 10 min at 4°C and decanted to remove cells, visually inspected for blood contamination, aliquoted in 1 mL cryotubes and stored at -70°C until analysis.

### CSF analysis

The 47 protein panel (S2 Table) for neurological biomarker discovery was based on our previous multiplex PLA panel [6] and selected according to broad neuropathologic or neurotherapeutic relevance. This lead to a panel of inflammatory mediators (interleukins, cytokines, chemokines), neurotrophic factors, glial markers, cell cycle regulators, adhesion proteins, soluble receptors or as enzymes or modulators of such proteins. Preference was given to targets with reported involvement in ALS or pain pathology (S1 Appendix), but technical aspects and assay performance were also taken into consideration. PSA was included in the panel as a control marker since it is known to differ between women and men. For a more detailed description on the panel protein selection see S1 Appendix.

#### Preparation of capture beads and PLA probes

Fifty μg of each antibody (S2 Table) was divided into one 10 μg aliquot and two aliquots of 20 μg. The 10 μg aliquot was immobilized on 2 mg of M-270 Epoxy Dynabeads using the antibody coupling kit (Life Technologies) according to the manufacturer’s protocol. The two 20 μg aliquots were used to prepare PLA probes (antibodies conjugated to oligonucleotides). The antibodies (2 mg/mL in PBS) were activated by adding 1 μL 4 mM sulfosuccinimidyl-4- (N-maleimidomethyl cyclohexane-1-carboxylate (sulfo-SMCC; Thermo Scientific) in DMSO (Sigma-Aldrich), and incubating at room temperature (RT) for 2 h. Oligonucleotides (S2 Table) for conjugation via sulphhydryl groups (15 μl, 20 μM)–purchased separately from IDT and Eurogentec to avoid contamination risks–were reduced by adding 15 μL of 50 mM DTT (Sigma-Aldrich) in 2xPBS with 5 mM EDTA (Sigma-Aldrich) and incubating at 37°C for 1 h. The SMCC activated antibodies and the reduced oligonucleotides were purified separately using Zeba Spin desalting plates, 7 K MWCO (Thermo Scientific) according to the manufacturer’s protocol. Each antibody preparation was mixed in separate reactions with each of the two oligonucleotides, and incubated at RT for 1.5 h, followed by dialysis overnight at 4°C against 5 l PBS with constant stirring by a magnetic bar in a 25 kD dialysis plate (Harvard Apparatus). The dialyzed antibody-oligonucleotide conjugates were diluted to 500 nM in PBS. Capture beads and PLA probes were stored at 4°C.

#### Multiplex SP-PLA

Twenty μL CSF samples were diluted by adding 25 μL PLA buffer (1 mM D-biotin (Invitrogen), 0.1% purified BSA (New England Biolabs), 0.05% Tween 20 (Sigma-Aldrich), 100 nM goat IgG (Sigma-Aldrich), 0.1 g/l salmon sperm DNA (Life Technologies), 5 mM EDTA, PBS), containing 5 pM mouse IgG (as a positive control). For purposes of methods evaluation, each sample was run in 4 technical replicates consuming a total of 80 μL of CSF. To make standard curves for quantification and determination limits of detection (LOD), solutions containing all 47 proteins were diluted in 10-fold steps from 500 pM to 5 fM in PLA buffer containing 5 pM mouse IgG, with one negative control (with none of the 47 proteins, denoted “blank”). Capture beads for all target proteins were combined, and 2.4 μL comprising 0.05 μL of each bead, was incubated with 45 μL of the diluted patient samples or with the dilutions of recombinant proteins. Reactions were incubated at RT for 1.5 h on a rotator. The beads were then washed once with washing buffer (PBS with 0.05% Tween 20), and mixed with 50 μL PLA buffer containing the PLA probes (500 pM of each) and incubated at RT for 1.5 h on a rotator. After washing, 50 μL ligation mix (1x ampligase buffer (Epicentre Biotechnology), 100 nM connecting oligo, and 0.02 U/μl ampligase (Epicentre Biotechnology)) was added, and the mixture was incubated at 37°C for 15 min. The microparticles were washed once and 50 μL PCR mix (1xPCR buffer (Invitrogen), 3 mM MgCl_2_ (Invitrogen), 0.2 mM d(A,U,G,C)TP mix (Fermentas), universal primers (S2 Table) 1 and 2 (100 nM each), 0.03 U/μl Platinum Taq DNA polymerase, 0.01 U/μl uracil-N-glycosylase (Fermentas)) was added to each reaction well. The ligation products were then amplified by PCR using the following program: 95°C for 10 min, followed by 15 cycles of 95°C for 15 sec, 62°C for 1 min, 72°C for 1min, and a final incubation at 8°C. The universal PCR product for each sample was diluted 25-fold into a new PCR mix (2xPCR buffer, 4 mM MgCl_2_, 0.4 mM d(A,U,G,C)TP, 1xSYBR Green, 0.06U/μl Taq DNA polymerase). Then 5 μL of each mixture was aliquoted into a 384-well plate in which each well was pre-spotted with 5 μL of a specific primer pair (S2 Table) for amplifying ligation products from each one of the protein detection reagents (200 nM of each primer) in water. The real time PCR was performed as 1 cycle at 95°C for 2 min, followed by 40 cycles of 95°C for 15 sec and 60°C for 1 min using an ABI-7900 instrument (Life Technologies).

#### Data analysis

A five parameter log-logistic function was fitted to the standard curve measurements, after outliers had been removed in a procedure based on Grubb's [39] test (for details see S2 Appendix). The LOD was defined as the protein concentration in the fitted standard curve that corresponded to the PCR cycle threshold m_Ctblank_—2s_Ctblank_ where m_Ctblank_ and s_Ctblank_ denote the mean and the standard deviation for threshold cycle (Ct) for the blank, respectively. The Ct is the threshold cycle, the real time PCR fractional cycle, where fluorescence reaches a preset threshold.

The variation of replicate samples was expressed as the robust coefficient of variation (robust CV), defined as the median absolute deviation divided by the median value for the replicate measurements.

As the data were not normally distributed, non-parametric testing was used. Ct values for each protein were compared between the ALS group and the control group using the Mann-Whitney U-test. To account for multiple comparisons, p-values less than 0.001 (significance level 0.05, Bonferroni correction 0.05/47≈0.001) were considered significant. The fold difference of patient sample to control was calculated as 2^-ΔCt^ (ΔCt = median (Ct_patient_)-median(Ct_control_)) where median(x) denotes the median of the individual values in the list x, one for each patient sample. The samples from patients with neuropathic pain were compared using Wilcoxon signed-rank test, which is the paired version of the Mann-Whitney U test. To investigate whether the level of any protein marker was influenced by the sex of the patients, measurements of all 47 proteins were compared between males (n = 20) and females (n = 20) in the ALS patients and controls using Mann-Whitney U test. All reproducible data were included in the statistical analyses, including values below the LOD calculated for the recombinant proteins in the standard curves.

To further evaluate how well the four identified ALS biomarker candidates could distinguish between ALS patient and control samples, multivariate prediction models were built using random forests [40]. The models were built through a repeated holdout procedure including a step where variables were selected based on Mann-Whitney U test statistics (for a detailed description see S2 Appendix).

## Results

### Assay performance

Of the 47 standard curves, all except interleukin-18 (IL-18) displayed a clear dose-response (S2 Fig) with a median dynamic range of 4 orders of magnitude and a median LOD of 2 pg/mL.

### Assessment of CSF proteins levels in 72 neurologically healthy controls

**Fig 1:**
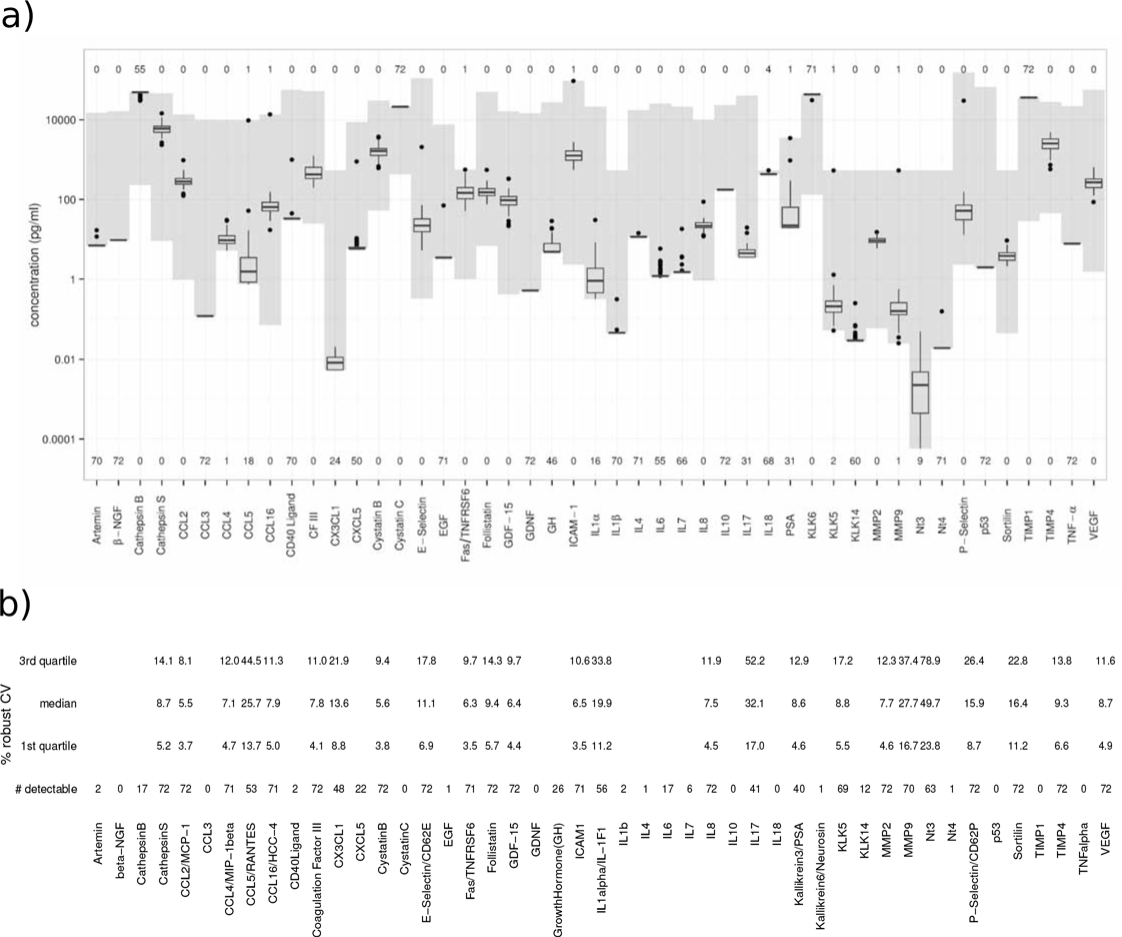
Protein variation among the investigated individuals. **A) Boxplots showing protein concentrations ranges in CSF from 72 individuals without neurological disorders.** The concentration ranges between the upper and lower limits of detection for each marker are shown in grey. The numbers below and above the boxplots show the number of patient samples (out of the total 72) that are outside the detection limits. **B) Performance measures for each protein assay.** The 1^st^, 2^nd^ (median) and 3^rd^ quartile values of the robust % CV. The numbers of detectable samples out of a total of 72 samples are found at the bottom line for each marker.

Of the 47 investigated proteins, 19 of them (cathepsin S, CCL2, CCL4, CCL16, CFIII, cystatin B, E-selectin, Fas/TNFRSF6, follistatin, GDF-15, ICAM-1, IL8, KLK5, MMP2, MMP9, P-selectin, sortilin, TIMP4 and VEGF) were detectable by multiplex SP-PLA in more than 95% (69) of the 72 control samples. The concentration ranges and the average CV of all samples for the 19 proteins are shown in Fig 1. For all 72 CSF samples a majority (15) of the protein measurements had an average CV below 10% (Fig 1). For four of the proteins the average CV values were higher (E-selectin (11.1%), P-selectin (15.9%), sortilin (16.4%) and MMP9 (27.7%)).

### Comparison of CSF protein levels in samples from ALS patients and controls

**Fig 2:**
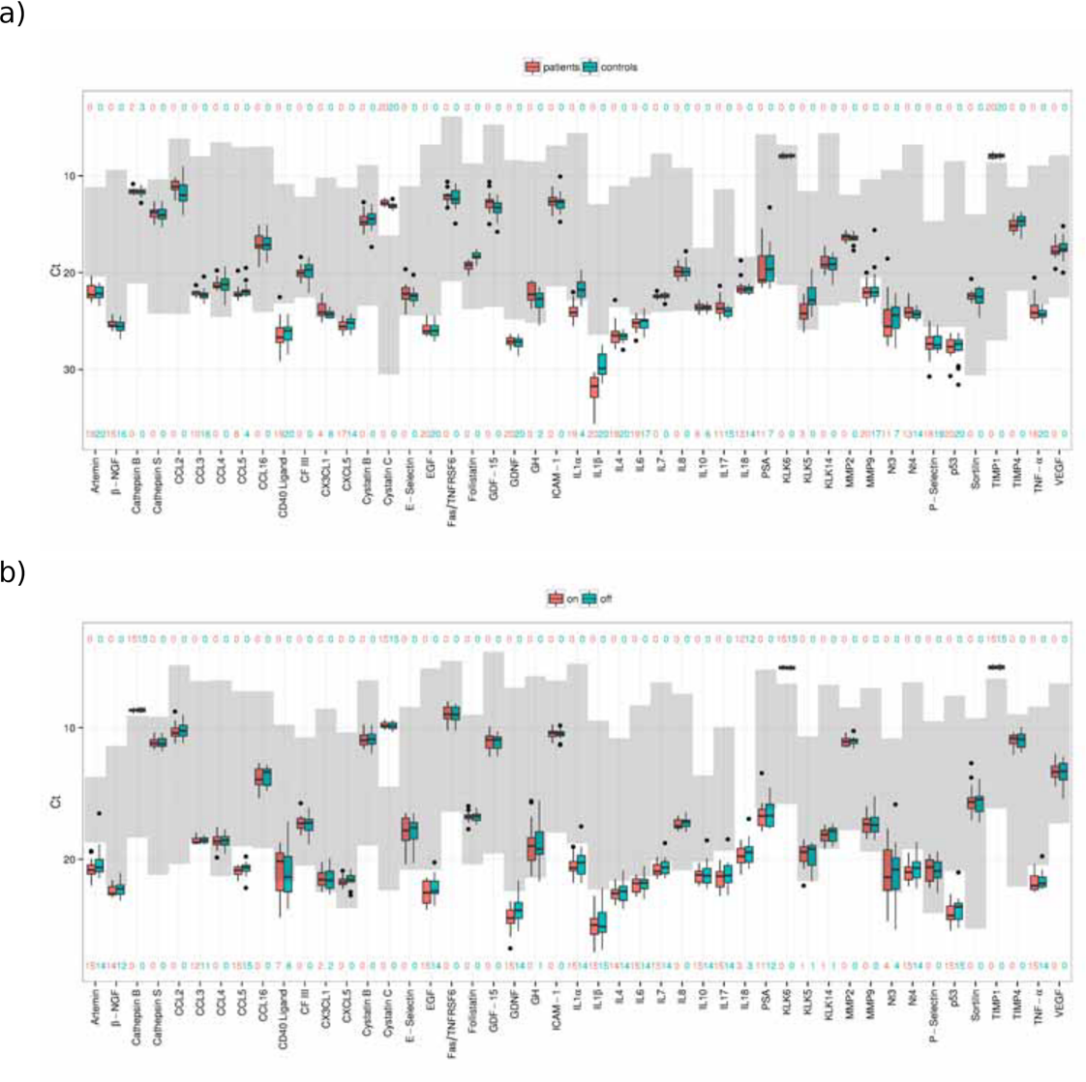
**Protein level comparisons for ALS patients and matched controls (a) and neuropathic pain patients with SCS on and off (b).** The y-axes represent Ct-values. The detectable concentration ranges between the upper and lower limits of detection for each marker are shown in grey. The numbers above and below the boxplots show the number of samples that are outside the detection limits.

**Fig 3:**
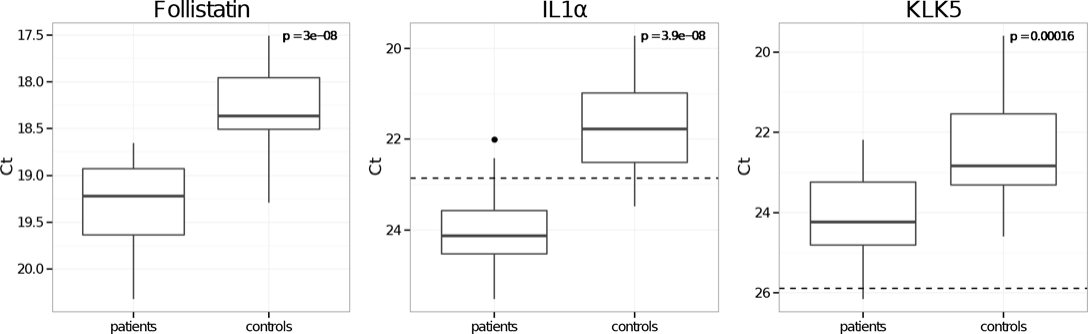
ALS Biomarker candidates. Boxplots showing the CSF levels for follistatin, IL1-alpha and KLK5 for ALS patients and matched controls. The protein levels are compared using the Mann-Whitney U-test and p-values are displayed in the Figures. The limits of detection are indicated by dashed horizontal lines. The y-axes represent Ct-values.

Of the 47 investigated proteins, 20 (cathepsin S, CCL2, CCL4, CCL16, CFIII, cystatin B, E-selectin, Fas/TNFRSF6, follistatin, GDF-15, GH, ICAM-1, IL7, IL8, KLK6, KLK14, MMP2, sortilin, TIMP4 and VEGF) were detectable by multiplex SP-PLA in ≥ 95% (39) of the ALS and control samples (Fig 2A). Four proteins, follistatin, interleukin-1 alpha (IL-1 alpha), interleukin-1 beta (IL-1 beta) and kallikrein-5 (KLK5), were found at significantly lower levels in the ALS samples than in control samples (p<0.001) (Table 1 and Fig 3), but IL-1beta was below LOD. Follistatin measurements were above LOD in all samples. KLK5 was above LOD in all control samples, and in 85% of the patient samples. IL-1 alpha was above LOD in 80% of the control samples, and in 5% of the patient samples.

**Table 1:** Results from univariate and multivariate analysis of ALS patients compared to controls.

Marker	p-value (Mann-Whitney U test)	Fold difference (patients/controls)	Mean AUC	Mean accuracy	Mean PD	Mean PFA	Fraction selected	Permutation p-value
Multivariate			0.95	87%	84%	10%		
Follistatin	3.0x10^-8^	55%	0.91	81%	83%	21%	1	<0.001
IL1-alpha	3.9x10^-8^	20%	0.89	72%	76%	32%	0.93	0.0090
IL1-beta	1.3x10^-7^	28%	0.93	85%	86%	16%	0.91	0.0060
KLK5	1.6x10^-4^	38%	0.67	54%	50%	41%	0.75	0.032
Cystatin C	0.0026	121%	0.65	59%	66%	48%	0.70	0.031
GH	0.021	137%	0.62	52%	54%	51%	0.42	0.12
CCL5	0.056	82%	0.49	52%	45%	40%	0.19	0.18
CCL2	0.063	184%	0.70	68%	69%	34%	0.27	0.12
GDF-15	0.063	149%	0.50	44%	45%	57%	0.24	0.14
TIMP4	0.063	71%	0.53	48%	48%	52%	0.14	0.25
CCL3	0.068	125%	0.54	46%	54%	61%	0.28	0.10
CX3CL1	0.10	115%	0.69	60%	57%	36%	0.14	0.29
IL17	0.10	116%	0.56	50%	53%	54%	0.25	0.15
CXCL5	0.16	76%	0.42	44%	38%	51%	0.01	0.73
beta-NGF	0.19	104%	0.41	44%	46%	58%	0.05	0.43
VEGF	0.23	84%	0.50	49%	56%	59%	0.01	0.66
E-Selectin	0.30	114%	0.39	41%	40%	58%	0.01	0.79
Nt3	0.34	45%	0.49	50%	46%	47%	0	1
CFIII	0.35	80%	0.58	56%	59%	47%	0.01	0.63
Nt4	0.37	115%	0.42	39%	38%	59%	0	1
GDNF	0.41	102%	0.41	45%	42%	52%	0	1
CD40Ligand	0.43	63%	0.53	57%	53%	40%	0	1
TNFRSF6	0.44	129%	0.53	48%	48%	52%	0	1
ICAM1	0.49	104%	0.67	63%	65%	40%	0	1
TNF-alpha	0.51	113%	0.53	53%	58%	53%	0	1
EGF	0.55	96%	0.42	40%	43%	62%	0	1
Cystatin B	0.60	78%	0.53	59%	55%	37%	0	1
PSA	0.60	46%	0.37	40%	45%	65%	0	1
MMP2	0.60	111%	0.42	50%	49%	49%	0	1
IL6	0.62	85%	0.52	45%	40%	50%	0	1
p53	0.64	81%	0.53	55%	53%	44%	0	1
Cathepsin S	0.66	123%	0.43	45%	51%	61%	0	1
KLK14	0.66	96%	0.67	62%	63%	38%	0	1
MMP9	0.72	100%	0.35	39%	33%	55%	0	1
IL18	0.74	101%	0.41	43%	42%	56%	0	1
CCL4	0.80	86%	0.64	64%	66%	39%	0	1
IL10	0.80	101%	0.39	49%	49%	51%	0	1
IL4	0.80	105%	0.48	54%	55%	47%	0	1
KLK6	0.80	98%	0.47	53%	59%	53%	0	1
IL8	0.82	101%	0.30	33%	40%	75%	0	1
IL7	0.86	100%	0.46	54%	49%	42%	0	1
Cathepsin B	0.88	100%	0.58	55%	58%	47%	0	1
Sortilin	0.90	108%	0.64	62%	65%	41%	0	1
TIMP1	0.95	99%	0.38	36%	43%	70%	0	1
Artemin	0.97	92%	0.28	35%	45%	75%	0	1
CCL16	0.97	93%	0.39	41%	36%	54%	0	1
P-Selectin	0.99	109%	0.24	35%	31%	62%	0	1

Columns 1 and 2 indicate p-values and fold differences for each marker. Columns 3, 4, 5 and 6 show mean AUC, mean accuracy, mean probability of detection (PD) (sensitivity) and probability of false alarm (PFA) (1-specificity) respectively for both the multivariate model and for the univariate models. Columns 7 and 8 reflect the variable importance for each marker (i.e. the fraction of models in which the particular variable was included) and the corresponding permutation p-values.

The multivariate prediction models had a mean accuracy of 0.87 (over all holdouts) and a mean area under the curve (AUC) of 0.95. The mean probability of detection (PD), i.e. classifying a patient as a patient, is 84% and the mean probability of false alarm (PFA), i.e. classifying a control as a patient, is 10%. The best single variable models were for IL-1 beta, follistatin and IL-1 alpha with mean AUC, accuracy, PD and PFA; 0.93, 0.85, 0.86, 0.16 (IL-1 beta); 0.91, 0.81, 0.83, 0.21 (follistatin); and 0.89, 0.72, 0.76, 0.32 (IL-1 alpha), respectively. Hence, the performances of the best single variable models were on the same level as the multivariate model. Performance measures for all markers are given in Table 1.

Among the four proteins with significantly lower levels in CSF from ALS patients compared to normal controls, follistatin had the lowest p-value, a high PD and low PFA as well as showing importance in the multivariate model. As expected levels of prostate specific antigen (PSA) differed significantly between males and females, both in ALS samples and their age-matched controls (p<1×10^−10^), but no other markers exhibited sex-dependent differences in expression.

### Comparison of CSF protein levels in samples from neuropathic patients with SCS on *vs*. off

Of the 47 investigated proteins, 21 (cathepsin S, CCL2, CCL4, CCL16, CFIII, CXCL5, cystatin B, E-Selectin, Fas/TNFRSF6, follistatin, GDF-15, GH, ICAM1, IL8, KLK5, MMP2, MMP9, P-Selectin, sortilin, TIMP4 and VEGF) were detectable by multiplex SP-PLA in ≥ 95% of the neuropathic pain patient samples. In addition, CX3CL1 (fractalkine) was detectable in ≥80% of the samples and Nt-3 was detectable in ≥50% of the samples. We found no significant differences in protein levels measured in the CSF of neuropathic pain patients between the SCS stimulated and non-stimulated condition (Fig 2B).

## Discussion

Firstly, we report decreased levels of follistatin, IL-1 alpha and KLK5 in CSF from ALS patients compared to controls. Secondly, we report unaltered levels of 21 proteins in neuropathic patients using SCS. Thirdly, we present 19 protein control concentration values, four of which (sortilin, CCL16, cystatin B, KLK5) have not, to our knowledge, previously been reported for adult individuals without neurological disease. We thereby illustrate the utility of multiplex SP-PLA panels as tools for neurological biomarker research.

Ethical and practical considerations restrict CSF sampling from healthy and neurologically healthy subjects [1, 41–43]. We provide 19 protein concentration ranges from 72 individuals scheduled for minor urological procedures, without acute infections, or neurological symptoms. This may provide a useful reference interval in the growing bank of published normal ranges of CSF protein concentrations.

Previous efforts to find biomarkers in CSF from ALS patients [44, 45] have identified glial proteins that correlate with survival time [44], and revealed increased levels of several proteins in ALS patients compared to neurological controls [45]. Follistatin [46, 47] is a multifunctional protein [48] that inhibits tissue remodeling, actions, and formation of fibrosis by binding activin A [49, 50] during inflammatory responses. Furthermore, follistatin treatment improves mean survival in another spinal motor neuron degenerative disease, spinal muscular atrophy (SMA) [51], probably by rescuing skeletal muscle. It is possible that the relatively lower level of follistatin we observe in the ALS patient CSF is a reflection of a generally lower follistatin concentration also in their blood and skeletal muscle tissue. On the other hand levels of follistatin mRNA have been shown to be overexpressed in skeletal muscle biopsies from a group of three ALS patients [52]. Activin A has been identified as a neuroprotective factor in nervous tissue and cell cultures [53–55].

IL1 (both alpha and beta) are produced by astrocytes [56–58], microglia [59–61], oligodendrocytes [62] and neurons [63–65] of the CNS, and can trigger responses in the same cell types [62, 66–76]. IL1 can have a protective or deleterious effect in the CNS [76]. For instance, IL1-beta has been shown to protect against oxidative stress [77]. The molecular protein pathology of ALS has earlier been studied using various proteomic approaches [78, 79]. Both follistatin [52, 80] and inflammation have previously been implicated in the pathology of ALS [45, 81, 82]. However, clinical trials using anti-inflammatory drugs against ALS have failed [83, 84], and may even have aggravated disease progression [85]. Here we show decreased levels of IL-1 alpha, which points to a possible exhaustion of these inflammatory mediators rather than an upregulation. A recent investigation of spinal microglia in a rat model of ALS found a previously unusual microglial phenotype with down-regulated expression of inflammatory mediators including TNF-alpha and IL-6 [24] (the level of these proteins were below the LOD in our assays). These two findings together with the failure of clinical trials targeting neuroinflammation in ALS [83, 86] point to the interesting hypothesis that ALS involves a reduction or imbalance in the microglial inflammatory response rather than an increase in inflammation, which would be in agreement with our findings. Recently several reports have highlighted the temporal [87] and spatial [24, 88] complexity of ALS neuroinflammation. Although studies give what appears to be contradictory results regarding the role of inflammation in ALS, we hope our data will contribute to clarification and progress in ALS mechanism research.

KLK5 expression has been found to be decreased in the gray matter of post-mortem human tissue after spinal cord injury [25, 89]. The decrease levels of KLK5 may serve as a marker for ongoing destruction and repair in the insulted nervous tissue. Although IL-1 beta was consistently found at levels below the LOD in all samples, the differences between the levels of IL-1 beta in patient- and control samples were statistically significant (p<1.3×10^−7^). In view of possible differences between human samples and those used for generating standard curves with recombinant *E*. *coli*-produced proteins and buffers for dilution we consider that the lower IL-1 beta levels found in patients may be meaningful, despite being below the LOD.

The levels of mRNA and secreted protein have been reported to be reduced for follistatin in peripheral blood mononuclear cells of relapsing-remitting multiple sclerosis (MS) patients [89], while the CSF level of follistatin protein is increased during meningitis [90]. Polymorphisms in IL-1alpha have been associated with increased risk of MS as well as its clinical course [91, 92]. KLK5 is a member of the kallikrein subgroup of the serine protease family of enzymes found in many different tissues including brain. Their roles in CNS are being mapped and they have been described as promising drug targets [93, 94].

Although cathepsin S [95], CCL2 [96], CCL4 [97], CFIII [98], E-Selectin [99], ICAM1 [100], IL8 [101], MMP2 and MMP9 [102], P-Selectin [103], sortilin [104], VEGF [105], CX3CL1 [106] and Nt-3 [107] have been implicated in neuropathic pain pathophysiology, our results suggest that SCS most likely does not change the CSF concentrations of these markers. It cannot be excluded that the pathways of these proteins may nonetheless be involved, since altered levels in the brain or spinal cord need not be reflected in CSF, and changes in post-translational modifications–which do not necessarily alter the affinity of the antibodies for the protein–would probably not be picked up by our assay. Another possible explanation for the negative result is that the time period of 48 hours when the SCS was turned off prior to sample collection–which also might bring severe pain for the patients–was not long enough to record any possible changes in the levels of protein concentrations. However, other studies showed increased VEGF levels in CSF after only 5 minutes of SCS in nine patients with failed back surgery syndrome (FBSS) [108]. The discrepancy between the results may be due to the differences in study designs and the patient populations, or perhaps there is an initial release of VEGF in CSF in response to SCS but levels stabilize overtime.

Conclusions from our findings must necessarily be tempered due to the modest sample sizes. In the case of the SCS patients results are strengthened by the opportunity for intra-individual comparisons, reducing influence by genetic or environmental influences that may account for some differences between individuals [109]. It should also be noted that the controls here were not healthy individuals, but they did lack manifest neurological symptoms or neurological disease as samples were collected in anaesthesia before urologic surgery. We therefore find it reasonable to assume that consistent differences in CSF protein levels compared to those of individuals with manifest ALS may in deed be related to ALS disease. There was a considerable overrepresentation of males in the reference cohort. However, no differences in protein level were observed in comparisons between males and females with the expected exception for PSA, indicating that CSF concentrations are not sex specific for any of the other investigated proteins.

In conclusion, by applying for the first time a 47-plex SP-PLA panel in analysis of CSF samples from individuals with neurological disease, we identified four ALS biomarker candidates. We demonstrated unchanged levels of 19 relevant CSF proteins during SCS treatment of neuropathic pain, and we report reference values for neurologically healthy controls. Further investigations are required to confirm or refute the potential value of decreased follistatin, IL1-alpha and KLK5 CSF levels as biomarkers of ALS disease. Our results illustrate the potential of multiplex SP-PLA and versions of these panel technologies as tools for neurological biomarker investigations.

## Supporting Information

S1 Appendix Description and rational behind selection of the protein panel. (PDF)

S2 Appendix Multivariate analysis. (PDF)

S1 Fig Flowchart of multiplex SP-PLA. I) Samples are incubated with a mixture of magnetic beads, each equipped with one immobilized capture antibody against one of the 47 target proteins. II) After washing, the mixture is incubated with 47 pairs of PLA probes (antibodies with conjugated DNA strands). III) After a second wash, a ligation reaction is performed in the presence of a connector DNA oligonucleotide to allow enzymatic joining of the two PLA probes to form a new amplifiable DNA molecule. IV) After a third washing step, PCR is performed using a pair of universal PCR primers. V) The PCR products are then diluted in a new PCR mix and aliquoted to 47 wells in a 384-well plate, each well pre-spotted with a specific pair of PCR primers for each investigated protein, and VI) real time PCR is performed and the Ct values are collected for data analysis.
(PDF)

S2 Fig Standard curves from analyses of 72 control samples for all proteins in the multiplex PLA panel. The fitted standard curve is shown in red and the values used to fit the standard curve are shown as black circles, the filled black circles are the measurements used as blanks. The LODs are shown as a cyan line and the LOD Ct-value is printed in the plots. The x-axis is shown in log-scale. Values along the x and y axes are indicated at the bottom and far left of the figure, respectively.
(PDF)

S1 Table Characteristics of clinical samples. (PDF)

S2 Table Antibodies and DNA oligonucleotides. (PDF)

## References

[1] Schutzer SE, Liu T, Natelson BH, Angel TE, Schepmoes AA, Purvine SO, et al Establishing the proteome of normal human cerebrospinal fluid. PLOS One. 2010;5(6):e10980 10.1371/journal.pone.0010980 20552007 PMC2881861

[2] Darmanis S, Yuan Nong R, Hammond M, Gu J, Alderborn A, Vänelid J, et al Sensitive plasma protein analysis by microparticle-based proximity ligation assays. Mol Cell Proteomics. 2009 . 19955079 10.1074/mcp.M900248-MCP200 PMC2830843

[3] Lundberg M, Thorsen SB, Assarsson E, Villablanca A, Tran B, Gee N, et al Multiplexed homogeneous proximity ligation assays for high-throughput protein biomarker research in serological material. Mol Cell Proteomics. 2011;10(4):M110.004978 10.1074/mcp.M110.004978 21242282 PMC3069344

[4] Fredriksson S, Horecka J, Brustugun O, Schlingemann J, Koong A, Tibshirani R, et al Multiplexed proximity ligation assays to profile putative plasma biomarkers relevant to pancreatic and ovarian cancer. Clin Chem. 2008;54(3):582–9. 10.1373/clinchem.2007.093195 18171715

[5] Darmanis S, Nong RY, Hammond M, Gu J, Alderborn A, Vänelid J, et al Sensitive plasma protein analysis by microparticle-based proximity ligation assays. Mol Cell Proteomics. 2010;9(2):327–35. 10.1074/mcp.M900248-MCP200 19955079 PMC2830843

[6] Darmanis S, Nong RY, Vänelid J, Siegbahn A, Ericsson O, Fredriksson S, et al ProteinSeq: high-performance proteomic analyses by proximity ligation and next generation sequencing. PLOS One. 2011;6(9):e25583 10.1371/journal.pone.0025583 21980495 PMC3183061

[7] Tavoosidana G, Ronquist G, Darmanis S, Yan J, Carlsson L, Wu D, et al Multiple recognition assay reveals prostasomes as promising plasma biomarkers for prostate cancer. Proc Natl Acad Sci U S A. 2011;108(21):8809–14. 10.1073/pnas.1019330108 21555566 PMC3102389

[8] Fredriksson S, Dixon W, Ji H, Koong A, Mindrinos M, Davis R. Multiplexed protein detection by proximity ligation for cancer biomarker validation. Nat Methods. 2007;4(4):327–9. doi: nmeth1020 [pii] 10.1038/nmeth1020 . 17369836

[9] van Hecke O, Austin SK, Khan RA, Smith BH, Torrance N. Neuropathic pain in the general population: A systematic review of epidemiological studies. Pain. 2013 10.1016/j.pain.2013.11.013 . 24291734

[10] Truin M, Janssen SP, van Kleef M, Joosten EA. Successful pain relief in non-responders to spinal cord stimulation: the combined use of ketamine and spinal cord stimulation. Eur J Pain. 2011;15(10):1049e1-9. 10.1016/j.ejpain.2011.04.004 . 21565537

[11] Pluijms WA, Slangen R, Bakkers M, Faber CG, Merkies IS, Kessels AG, et al Pain relief and quality-of-life improvement after spinal cord stimulation in painful diabetic polyneuropathy: a pilot study. Br J Anaesth. 2012;109(4):623–9. 10.1093/bja/aes251 . 22893671

[12] Ohnmeiss DD, Rashbaum RF, Bogdanffy GM. Prospective outcome evaluation of spinal cord stimulation in patients with intractable leg pain. Spine (Phila Pa 1976). 1996;21(11):1344–50; discussion 51. . 8725927 10.1097/00007632-199606010-00013

[13] Spiegelmann R, Friedman WA. Spinal cord stimulation: a contemporary series. Neurosurgery. 1991;28(1):65–70; discussion -1. . 1704492

[14] Graves MC, Fiala M, Dinglasan LA, Liu NQ, Sayre J, Chiappelli F, et al Inflammation in amyotrophic lateral sclerosis spinal cord and brain is mediated by activated macrophages, mast cells and T cells. Amyotroph Lateral Scler Other Motor Neuron Disord. 2004;5(4):213–9. . 15799549 10.1080/14660820410020286

[15] Philips T, Robberecht W. Neuroinflammation in amyotrophic lateral sclerosis: role of glial activation in motor neuron disease. Lancet Neurol. 2011;10(3):253–63. 10.1016/S1474-4422(11)70015-1 . 21349440

[16] Robberecht W, Philips T. The changing scene of amyotrophic lateral sclerosis. Nat Rev Neurosci. 2013;14(4):248–64. 10.1038/nrn3430 . 23463272

[17] Noh MY, Cho KA, Kim H, Kim SM, Kim SH. Erythropoietin modulates the immune-inflammatory response of a SOD1(G93A) transgenic mouse model of amyotrophic lateral sclerosis (ALS). Neurosci Lett. 2014;574:53–8. 10.1016/j.neulet.2014.05.001 . 24820540

[18] Henkel JS, Beers DR, Zhao W, Appel SH. Microglia in ALS: the good, the bad, and the resting. J Neuroimmune Pharmacol. 2009;4(4):389–98. 10.1007/s11481-009-9171-5 . 19731042

[19] Yamanaka K, Chun SJ, Boillee S, Fujimori-Tonou N, Yamashita H, Gutmann DH, et al Astrocytes as determinants of disease progression in inherited amyotrophic lateral sclerosis. Nat Neurosci. 2008;11(3):251–3. 10.1038/nn2047 18246065 PMC3137510

[20] Boillée S, Vande Velde C, Cleveland DW. ALS: a disease of motor neurons and their nonneuronal neighbors. Neuron. 2006;52(1):39–59. 10.1016/j.neuron.2006.09.018 . 17015226

[21] Boillée S, Yamanaka K, Lobsiger CS, Copeland NG, Jenkins NA, Kassiotis G, et al Onset and progression in inherited ALS determined by motor neurons and microglia. Science. 2006;312(5778):1389–92. 10.1126/science.1123511 . 16741123

[22] Wang L, Sharma K, Grisotti G, Roos RP. The effect of mutant SOD1 dismutase activity on non-cell autonomous degeneration in familial amyotrophic lateral sclerosis. Neurobiol Dis. 2009;35(2):234–40. 10.1016/j.nbd.2009.05.002 19442735 PMC2706919

[23] Wang L, Gutmann DH, Roos RP. Astrocyte loss of mutant SOD1 delays ALS disease onset and progression in G85R transgenic mice. Hum Mol Genet. 2011;20(2):286–93. 10.1093/hmg/ddq463 . 20962037

[24] Nikodemova M, Small AL, Smith SM, Mitchell GS, Watters JJ. Spinal but not cortical microglia acquire an atypical phenotype with high VEGF, galectin-3 and osteopontin, and blunted inflammatory responses in ALS rats. Neurobiol Dis. 2014;69:43–53. 10.1016/j.nbd.2013.11.009 24269728 PMC4079765

[25] Mika J, Zychowska M, Popiolek-Barczyk K, Rojewska E, Przewlocka B. Importance of glial activation in neuropathic pain. Eur J Pharmacol. 2013;716(1–3):106–19. 10.1016/j.ejphar.2013.01.072 . 23500198

[26] Scholz J, Woolf CJ. The neuropathic pain triad: neurons, immune cells and glia. Nat Neurosci. 2007;10(11):1361–8. 10.1038/nn1992 . 17965656

[27] Austin PJ, Moalem-Taylor G. The neuro-immune balance in neuropathic pain: involvement of inflammatory immune cells, immune-like glial cells and cytokines. J Neuroimmunol. 2010;229(1–2):26–50. 10.1016/j.jneuroim.2010.08.013 . 20870295

[28] Tsuda M, Inoue K. Neuron-microglia interaction by purinergic signaling in neuropathic pain following neurodegeneration. Neuropharmacology. 2015 10.1016/j.neuropharm.2015.08.042 . 26327676

[29] Tsuda M, Inoue K, Salter MW. Neuropathic pain and spinal microglia: a big problem from molecules in "small" glia. Trends Neurosci. 2005;28(2):101–7. 10.1016/j.tins.2004.12.002 . 15667933

[30] Tsuda M, Beggs S, Salter MW, Inoue K. Microglia and intractable chronic pain. Glia. 2013;61(1):55–61. 10.1002/glia.22379 . 22740331

[31] McMahon SB, Malcangio M. Current challenges in glia-pain biology. Neuron. 2009;64(1):46–54. 10.1016/j.neuron.2009.09.033 . 19840548

[32] DeVon HA, Piano MR, Rosenfeld AG, Hoppensteadt DA. The association of pain with protein inflammatory biomarkers: a review of the literature. Nurs Res. 2014;63(1):51–62. 10.1097/NNR.0000000000000013 . 24335913

[33] Nijs J, Meeus M, Versijpt J, Moens M, Bos I, Knaepen K, et al Brain-derived neurotrophic factor as a driving force behind neuroplasticity in neuropathic and central sensitization pain: a new therapeutic target? Expert Opin Ther Targets. 2015;19(4):565–76. 10.1517/14728222.2014.994506 . 25519921

[34] McMahon SB, Jones NG. Plasticity of pain signaling: role of neurotrophic factors exemplified by acid-induced pain. J Neurobiol. 2004;61(1):72–87. 10.1002/neu.20093 . 15362154

[35] Pezet S, McMahon SB. Neurotrophins: mediators and modulators of pain. Annu Rev Neurosci. 2006;29:507–38. 10.1146/annurev.neuro.29.051605.112929 . 16776595

[36] Wolf G, Gabay E, Tal M, Yirmiya R, Shavit Y. Genetic impairment of interleukin-1 signaling attenuates neuropathic pain, autotomy, and spontaneous ectopic neuronal activity, following nerve injury in mice. Pain. 2006;120(3):315–24. 10.1016/j.pain.2005.11.011 . 16426759

[37] Cunha TM, Verri WA, Silva JS, Poole S, Cunha FQ, Ferreira SH. A cascade of cytokines mediates mechanical inflammatory hypernociception in mice. Proc Natl Acad Sci U S A. 2005;102(5):1755–60. 10.1073/pnas.0409225102 15665080 PMC547882

[38] Schäfers M, Lee DH, Brors D, Yaksh TL, Sorkin LS. Increased sensitivity of injured and adjacent uninjured rat primary sensory neurons to exogenous tumor necrosis factor-alpha after spinal nerve ligation. J Neurosci. 2003;23(7):3028–38. . 12684490 10.1523/JNEUROSCI.23-07-03028.2003 PMC6742101

[39] Grubbs F. Procedures for detecting outlying observations in samples. Technometrics. 1969;11(1):1–21. 10.2307/1266761 .

[40] Breiman L. Random Forests. Machine Learning. 2001;45(1):5–32.

[41] Zhang J, Goodlett DR, Peskind ER, Quinn JF, Zhou Y, Wang Q, et al Quantitative proteomic analysis of age-related changes in human cerebrospinal fluid. Neurobiol Aging. 2005;26(2):207–27. 10.1016/j.neurobiolaging.2004.03.012 . 15582749

[42] Xu J, Chen J, Peskind ER, Jin J, Eng J, Pan C, et al Characterization of proteome of human cerebrospinal fluid. Int Rev Neurobiol. 2006;73:29–98. 10.1016/S0074-7742(06)73002-1 . 16737901

[43] Pan S, Zhu D, Quinn JF, Peskind ER, Montine TJ, Lin B, et al A combined dataset of human cerebrospinal fluid proteins identified by multi-dimensional chromatography and tandem mass spectrometry. Proteomics. 2007;7(3):469–73. 10.1002/pmic.200600756 . 17211832

[44] Süssmuth SD, Sperfeld AD, Hinz A, Brettschneider J, Endruhn S, Ludolph AC, et al CSF glial markers correlate with survival in amyotrophic lateral sclerosis. Neurology. 2010;74(12):982–7. 10.1212/WNL.0b013e3181d5dc3b . 20308682

[45] Mitchell RM, Freeman WM, Randazzo WT, Stephens HE, Beard JL, Simmons Z, et al A CSF biomarker panel for identification of patients with amyotrophic lateral sclerosis. Neurology. 2009;72(1):14–9. 10.1212/01.wnl.0000333251.36681.a5 . 18987350

[46] Esch FS, Shimasaki S, Mercado M, Cooksey K, Ling N, Ying S, et al Structural characterization of follistatin: a novel follicle-stimulating hormone release-inhibiting polypeptide from the gonad. Mol Endocrinol. 1987;1(11):849–55. 10.1210/mend-1-11-849 . 3153465

[47] Robertson DM, Klein R, de Vos FL, McLachlan RI, Wettenhall RE, Hearn MT, et al The isolation of polypeptides with FSH suppressing activity from bovine follicular fluid which are structurally different to inhibin. Biochem Biophys Res Commun. 1987;149(2):744–9. . 3122741 10.1016/0006-291x(87)90430-x

[48] Phillips DJ, de Kretser DM. Follistatin: a multifunctional regulatory protein. Front Neuroendocrinol. 1998;19(4):287–322. 10.1006/frne.1998.0169 . 9799587

[49] de Kretser DM, O'Hehir RE, Hardy CL, Hedger MP. The roles of activin A and its binding protein, follistatin, in inflammation and tissue repair. Mol Cell Endocrinol. 2012;359(1–2):101–6. 10.1016/j.mce.2011.10.009 . 22037168

[50] Nakamura T, Takio K, Eto Y, Shibai H, Titani K, Sugino H. Activin-binding protein from rat ovary is follistatin. Science. 1990;247(4944):836–8. . 2106159 10.1126/science.2106159

[51] Rose FF, Mattis VB, Rindt H, Lorson CL. Delivery of recombinant follistatin lessens disease severity in a mouse model of spinal muscular atrophy. Hum Mol Genet. 2009;18(6):997–1005. 10.1093/hmg/ddn426 19074460 PMC2649020

[52] Shtilbans A, Choi SG, Fowkes ME, Khitrov G, Shahbazi M, Ting J, et al Differential gene expression in patients with amyotrophic lateral sclerosis. Amyotroph Lateral Scler. 2011;12(4):250–6. 10.3109/17482968.2011.560946 . 21375368

[53] Iwahori Y, Saito H, Torii K, Nishiyama N. Activin exerts a neurotrophic effect on cultured hippocampal neurons. Brain Res. 1997;760(1–2):52–8. . 9237517 10.1016/s0006-8993(97)00275-8

[54] Schubert D, Kimura H, LaCorbiere M, Vaughan J, Karr D, Fischer WH. Activin is a nerve cell survival molecule. Nature. 1990;344(6269):868–70. 10.1038/344868a0 . 2330043

[55] Wu DD, Lai M, Hughes PE, Sirimanne E, Gluckman PD, Williams CE. Expression of the activin axis and neuronal rescue effects of recombinant activin A following hypoxic-ischemic brain injury in the infant rat. Brain Res. 1999;835(2):369–78. . 10415398 10.1016/s0006-8993(99)01638-8

[56] Knerlich F, Schilling L, Görlach C, Wahl M, Ehrenreich H, Sirén AL. Temporal profile of expression and cellular localization of inducible nitric oxide synthase, interleukin-1beta and interleukin converting enzyme after cryogenic lesion of the rat parietal cortex. Brain Res Mol Brain Res. 1999;68(1–2):73–87. . 10320785 10.1016/s0169-328x(99)00064-9

[57] Lieberman AP, Pitha PM, Shin HS, Shin ML. Production of tumor necrosis factor and other cytokines by astrocytes stimulated with lipopolysaccharide or a neurotropic virus. Proc Natl Acad Sci U S A. 1989;86(16):6348–52. 2474832 10.1073/pnas.86.16.6348 PMC297836

[58] Zhang W, Smith C, Howlett C, Stanimirovic D. Inflammatory activation of human brain endothelial cells by hypoxic astrocytes in vitro is mediated by IL-1beta. J Cereb Blood Flow Metab. 2000;20(6):967–78. 10.1097/00004647-200006000-00009 . 10894180

[59] Giulian D, Baker TJ, Shih LC, Lachman LB. Interleukin 1 of the central nervous system is produced by ameboid microglia. J Exp Med. 1986;164(2):594–604. 3487617 10.1084/jem.164.2.594 PMC2188228

[60] Hetier E, Ayala J, Denèfle P, Bousseau A, Rouget P, Mallat M, et al Brain macrophages synthesize interleukin-1 and interleukin-1 mRNAs in vitro. J Neurosci Res. 1988;21(2–4):391–7. 10.1002/jnr.490210230 . 3265161

[61] Yao J, Keri JE, Taffs RE, Colton CA. Characterization of interleukin-1 production by microglia in culture. Brain Res. 1992;591(1):88–93. . 1446236 10.1016/0006-8993(92)90981-e

[62] Blasi F, Riccio M, Brogi A, Strazza M, Taddei ML, Romagnoli S, et al Constitutive expression of interleukin-1beta (IL-1beta) in rat oligodendrocytes. Biol Chem. 1999;380(2):259–64. 10.1515/BC.1999.034 . 10195433

[63] Lechan RM, Toni R, Clark BD, Cannon JG, Shaw AR, Dinarello CA, et al Immunoreactive interleukin-1 beta localization in the rat forebrain. Brain Res. 1990;514(1):135–40. . 2357520 10.1016/0006-8993(90)90445-h

[64] Takao T, Tracey DE, Mitchell WM, De Souza EB. Interleukin-1 receptors in mouse brain: characterization and neuronal localization. Endocrinology. 1990;127(6):3070–8. 10.1210/endo-127-6-3070 . 2147409

[65] Watt JA, Hobbs NK. Interleukin-1beta immunoreactivity in identified neurons of the rat magnocellular neurosecretory system: evidence for activity-dependent release. J Neurosci Res. 2000;60(4):478–89. . 10797550 10.1002/(SICI)1097-4547(20000515)60:4<478::AID-JNR6>3.0.CO;2-R

[66] Ban E, Milon G, Prudhomme N, Fillion G, Haour F. Receptors for interleukin-1 (alpha and beta) in mouse brain: mapping and neuronal localization in hippocampus. Neuroscience. 1991;43(1):21–30. . 1833666 10.1016/0306-4522(91)90412-h

[67] Ban EM, Sarliève LL, Haour FG. Interleukin-1 binding sites on astrocytes. Neuroscience. 1993;52(3):725–33. . 8450969 10.1016/0306-4522(93)90421-b

[68] Cunningham ET, De Souza EB. Interleukin 1 receptors in the brain and endocrine tissues. Immunol Today. 1993;14(4):171–6. . 8499077 10.1016/0167-5699(93)90281-o

[69] French RA, VanHoy RW, Chizzonite R, Zachary JF, Dantzer R, Parnet P, et al Expression and localization of p80 and p68 interleukin-1 receptor proteins in the brain of adult mice. J Neuroimmunol. 1999;93(1–2):194–202. . 10378883 10.1016/s0165-5728(98)00224-0

[70] Friedman WJ. Cytokines regulate expression of the type 1 interleukin-1 receptor in rat hippocampal neurons and glia. Exp Neurol. 2001;168(1):23–31. 10.1006/exnr.2000.7595 . 11170718

[71] Hammond EA, Smart D, Toulmond S, Suman-Chauhan N, Hughes J, Hall MD. The interleukin-1 type I receptor is expressed in human hypothalamus. Brain. 1999;122 (Pt 9):1697–707. . 10468509 10.1093/brain/122.9.1697

[72] Pinteaux E, Parker LC, Rothwell NJ, Luheshi GN. Expression of interleukin-1 receptors and their role in interleukin-1 actions in murine microglial cells. J Neurochem. 2002;83(4):754–63. . 12421347 10.1046/j.1471-4159.2002.01184.x

[73] Tomozawa Y, Inoue T, Satoh M. Expression of type I interleukin-1 receptor mRNA and its regulation in cultured astrocytes. Neurosci Lett. 1995;195(1):57–60. . 7478255 10.1016/0304-3940(95)11781-q

[74] Wang XF, Yin L, Hu JG, Huang LD, Yu PP, Jiang XY, et al Expression and localization of p80 interleukin-1 receptor protein in the rat spinal cord. J Mol Neurosci. 2006;29(1):45–53. 10.1385/JMN:29:1:45 . 16757809

[75] Wong ML, Licinio J. Localization of interleukin 1 type I receptor mRNA in rat brain. Neuroimmunomodulation. 1994;1(2):110–5. . 7489320 10.1159/000097143

[76] Hewett SJ, Jackman NA, Claycomb RJ. Interleukin-1β in Central Nervous System Injury and Repair. Eur J Neurodegener Dis. 2012;1(2):195–211. 26082912 PMC4465544

[77] He Y, Jackman NA, Thorn TL, Vought VE, Hewett SJ. Interleukin-1β protects astrocytes against oxidant-induced injury via an NF-κB-dependent upregulation of glutathione synthesis. Glia. 2015;63(9):1568–80. 10.1002/glia.22828 25880604 PMC4506211

[78] Ramström M, Ivonin I, Johansson A, Askmark H, Markides KE, Zubarev R, et al Cerebrospinal fluid protein patterns in neurodegenerative disease revealed by liquid chromatography-Fourier transform ion cyclotron resonance mass spectrometry. Proteomics. 2004;4(12):4010–8. 10.1002/pmic.200400871 . 15540204

[79] Elf K, Shevchenko G, Nygren I, Larsson L, Bergquist J, Askmark H, et al Alterations in muscle proteome of patients diagnosed with amyotrophic lateral sclerosis. J Proteomics. 2014;108:55–64. 10.1016/j.jprot.2014.05.004 . 24846852

[80] Miller TM, Kim SH, Yamanaka K, Hester M, Umapathi P, Arnson H, et al Gene transfer demonstrates that muscle is not a primary target for non-cell-autonomous toxicity in familial amyotrophic lateral sclerosis. Proc Natl Acad Sci U S A. 2006;103(51):19546–51. 10.1073/pnas.0609411103 17164329 PMC1748262

[81] McCombe PA, Henderson RD. The Role of immune and inflammatory mechanisms in ALS. Curr Mol Med. 2011;11(3):246–54. 21375489 10.2174/156652411795243450 PMC3182412

[82] Sekizawa T, Openshaw H, Ohbo K, Sugamura K, Itoyama Y, Niland JC. Cerebrospinal fluid interleukin 6 in amyotrophic lateral sclerosis: immunological parameter and comparison with inflammatory and non-inflammatory central nervous system diseases. J Neurol Sci. 1998;154(2):194–9. . 9562310 10.1016/s0022-510x(97)00228-1

[83] Cudkowicz ME, Shefner JM, Schoenfeld DA, Zhang H, Andreasson KI, Rothstein JD, et al Trial of celecoxib in amyotrophic lateral sclerosis. Ann Neurol. 2006;60(1):22–31. 10.1002/ana.20903 . 16802291

[84] Werdelin L, Boysen G, Jensen TS, Mogensen P. Immunosuppressive treatment of patients with amyotrophic lateral sclerosis. Acta Neurol Scand. 1990;82(2):132–4. . 2256442 10.1111/j.1600-0404.1990.tb01602.x

[85] Gordon PH, Moore DH, Miller RG, Florence JM, Verheijde JL, Doorish C, et al Efficacy of minocycline in patients with amyotrophic lateral sclerosis: a phase III randomised trial. Lancet Neurol. 2007;6(12):1045–53. 10.1016/S1474-4422(07)70270-3 . 17980667

[86] Benatar M. Lost in translation: treatment trials in the SOD1 mouse and in human ALS. Neurobiol Dis. 2007;26(1):1–13. 10.1016/j.nbd.2006.12.015 . 17300945

[87] Hooten KG, Beers DR, Zhao W, Appel SH. Protective and Toxic Neuroinflammation in Amyotrophic Lateral Sclerosis. Neurotherapeutics. 2015;12(2):364–75. 10.1007/s13311-014-0329-3 25567201 PMC4404435

[88] Berjaoui S, Povedano M, Garcia-Esparcia P, Carmona M, Aso E, Ferrer I. Complex Inflammation mRNA-Related Response in ALS Is Region Dependent. Neural Plast. 2015;2015:573784 10.1155/2015/573784 26301107 PMC4537753

[89] Urshansky N, Mausner-Fainberg K, Auriel E, Regev K, Karni A. Low and dysregulated production of follistatin in immune cells of relapsing-remitting multiple sclerosis patients. J Neuroimmunol. 2011;238(1–2):96–103. 10.1016/j.jneuroim.2011.08.003 . 21880375

[90] Michel U, Ebert S, Schneider O, Shintani Y, Bunkowski S, Smirnov A, et al Follistatin (FS) in human cerebrospinal fluid and regulation of FS expression in a mouse model of meningitis. Eur J Endocrinol. 2000;143(6):809–16. . 11124865 10.1530/eje.0.1430809

[91] Mirowska-Guzel D, Gromadzka G, Mach A, Czlonkowski A, Czlonkowska A. Association of IL1A, IL1B, ILRN, IL6, IL10 and TNF-α polymorphisms with risk and clinical course of multiple sclerosis in a Polish population. J Neuroimmunol. 2011;236(1–2):87–92. 10.1016/j.jneuroim.2011.04.014 . 21621860

[92] Mann CL, Davies MB, Stevenson VL, Leary SM, Boggild MD, Ko Ko C, et al Interleukin 1 genotypes in multiple sclerosis and relationship to disease severity. J Neuroimmunol. 2002;129(1–2):197–204. . 12161036 10.1016/s0165-5728(02)00181-9

[93] Prassas I, Eissa A, Poda G, Diamandis EP. Unleashing the therapeutic potential of human kallikrein-related serine proteases. Nat Rev Drug Discov. 2015;14(3):183–202. 10.1038/nrd4534 . 25698643

[94] Yousef GM, Kishi T, Diamandis EP. Role of kallikrein enzymes in the central nervous system. Clin Chim Acta. 2003;329(1–2):1–8. . 12589961 10.1016/s0009-8981(03)00004-4

[95] Clark AK, Yip PK, Grist J, Gentry C, Staniland AA, Marchand F, et al Inhibition of spinal microglial cathepsin S for the reversal of neuropathic pain. Proc Natl Acad Sci U S A. 2007;104(25):10655–60. 10.1073/pnas.0610811104 17551020 PMC1965568

[96] Zhu X, Cao S, Zhu MD, Liu JQ, Chen JJ, Gao YJ. Contribution of Chemokine CCL2/CCR2 Signaling in the Dorsal Root Ganglion and Spinal Cord to the Maintenance of Neuropathic Pain in a Rat Model of Lumbar Disc Herniation. J Pain. 2014 10.1016/j.jpain.2014.01.492 . 24462503

[97] Saika F, Kiguchi N, Kobayashi Y, Fukazawa Y, Kishioka S. CC-chemokine ligand 4/macrophage inflammatory protein-1β participates in the induction of neuropathic pain after peripheral nerve injury. Eur J Pain. 2012;16(9):1271–80. 10.1002/j.1532-2149.2012.00146.x . 22528550

[98] Nie F, Wang J, Su D, Shi Y, Chen J, Wang H, et al Abnormal activation of complement C3 in the spinal dorsal horn is closely associated with progression of neuropathic pain. Int J Mol Med. 2013;31(6):1333–42. 10.3892/ijmm.2013.1344 . 23588254

[99] Tufan K, Sen O, Cekinmez M, Bolat FA, Alkan O, Sarica FB, et al Comparison of E-selectin and the other inflammatory markers in lumbar disc herniation: a new promising therapeutical window for radicular pain. J Spinal Disord Tech. 2012;25(8):443–6. 10.1097/BSD.0b013e318238e2db . 22015628

[100] Sweitzer SM, White KA, Dutta C, DeLeo JA. The differential role of spinal MHC class II and cellular adhesion molecules in peripheral inflammatory versus neuropathic pain in rodents. J Neuroimmunol. 2002;125(1–2):82–93. . 11960644 10.1016/s0165-5728(02)00036-x

[101] Kim SJ, Park SM, Cho YW, Jung YJ, Lee DG, Jang SH, et al Changes in expression of mRNA for interleukin-8 and effects of interleukin-8 receptor inhibitor in the spinal dorsal horn in a rat model of lumbar disc herniation. Spine (Phila Pa 1976). 2011;36(25):2139–46. 10.1097/BRS.0b013e31821945a3 . 21415806

[102] Kawasaki Y, Xu ZZ, Wang X, Park JY, Zhuang ZY, Tan PH, et al Distinct roles of matrix metalloproteases in the early- and late-phase development of neuropathic pain. Nat Med. 2008;14(3):331–6. 10.1038/nm1723 18264108 PMC2279180

[103] Liou JT, Lee CM, Lin YC, Chen CY, Liao CC, Lee HC, et al P-selectin is required for neutrophils and macrophage infiltration into injured site and contributes to generation of behavioral hypersensitivity following peripheral nerve injury in mice. Pain. 2013;154(10):2150–9. 10.1016/j.pain.2013.06.042 . 23831400

[104] Lewin GR, Nykjaer A. Pro-neurotrophins, sortilin, and nociception. Eur J Neurosci. 2014;39(3):363–74. 10.1111/ejn.12466 . 24494677 PMC4232910

[105] Kiguchi N, Kobayashi Y, Kadowaki Y, Fukazawa Y, Saika F, Kishioka S. Vascular endothelial growth factor signaling in injured nerves underlies peripheral sensitization in neuropathic pain. J Neurochem. 2014;129(1):169–78. 10.1111/jnc.12614 . 24304382

[106] Staniland AA, Clark AK, Wodarski R, Sasso O, Maione F, D'Acquisto F, et al Reduced inflammatory and neuropathic pain and decreased spinal microglial response in fractalkine receptor (CX3CR1) knockout mice. J Neurochem. 2010;114(4):1143–57. 10.1111/j.1471-4159.2010.06837.x . 20524966

[107] Richner M, Ulrichsen M, Elmegaard SL, Dieu R, Pallesen LT, Vaegter CB. Peripheral Nerve Injury Modulates Neurotrophin Signaling in the Peripheral and Central Nervous System. Mol Neurobiol. 2014 10.1007/s12035-014-8706-9 . 24752592

[108] McCarthy KF, Connor TJ, McCrory C. Cerebrospinal fluid levels of vascular endothelial growth factor correlate with reported pain and are reduced by spinal cord stimulation in patients with failed back surgery syndrome. Neuromodulation. 2013;16(6):519–22; discussion 22. 10.1111/j.1525-1403.2012.00527.x . 23136965

[109] Enroth S, Johansson A, Enroth SB, Gyllensten U. Strong effects of genetic and lifestyle factors on biomarker variation and use of personalized cutoffs. Nat Commun. 2014;5:4684 10.1038/ncomms5684 25147954 PMC4143927

