# Supplementary material for: A Multiplex Protein Panel Applied to Cerebrospinal Fluid Reveals Three New Biomarker Candidates in ALS but None in Neuropathic Pain Patients
Source: PLoS One. 2016 Feb 25;11(2):e0149821. doi: 10.1371/journal.pone.0149821 (PMC4767403; doi:10.1371/journal.pone.0149821)
Supplement: S1 Appendix — (PDF) [file pone.0149821.s001.pdf]

Appendix 1- PLA panel target selection

Our panel for neurological biomarker discovery was selected on the basis of the following criteria:

1. Broad neuropathologic or neurotherapeutic relevance (all selected markers are potentially of interest in neuropathologies for example as inflammatory mediators (interleukins, cytokines, chemokines), neurotrophic factors, glial markers, cell cycle regulators, adhesion proteins, soluble receptors or as enzymes or modulators of such proteins
2. Reports in the literature for involvement in ALS or pain pathology
3. Availability of suitable antibodies
4. Satisfactory dose-response to recombinant protein dilution series in pilot detection assays, indicating functional assays.
5. PSA was included in the panel as a control marker since it is known to differ between women and men.

Listed below are further details for each of the markers

1. Sortilin – pNGF co-receptor, pain, plasticity. Receptor for proNGF, which is investigated for a role in chronic pain signaling [1]. Mice lacking sortilin expression show reduced response to neurotrophins [2]. Hypothesized to be an independent receptor signaling system in neuropathic and inflammatory pain [1]. Has been investigated for a role in ALS, but no contributing mutation of its gene was found in that study [3]. Regulate the function and viability of neurons [4]. One of the genes regulated by ALS inclusion protein Tar DNA binding protein 43[5] .
2. GDNF – Glia-neuron, plasticity. Found to be increased in CSF of ALS patients [6]. Sensitizes nociceptors [7]. Pain was associated with levels of increased GDNF levels ub CSF and decreased levels in blood [8]. Potent survival factor for motor neurons with possible role in ALS treatment [9].
3. Artemin – Neurotrophic factor. GDNF family. Alters receptor subunit expression and activity in nociceptive neurons [10]. Sensitizes nociceptors [7]. Supports sympathetic and sensory neuron survival and is a candidate for treatment of chronic pain [11].
4. CCL2 – Induces central sensitization by increasing the activity of NMDA receptors in dorsal horn neurons [12]. Negatively correlated to functionality score in CSF of ALS patients [13]. Upregulated upon disease onset in mouse ALS model [14]. Regulates macrophage response after nerve injury [15]. Contributes to central sensitization and neuropathic pain via spinal cord astrocytes [16]. Involved in the maintenance of neuropathic pain in a rat model of lumbar radicular pain [17]. Decreased after SCS in an animal model of neuropathic pain [18].
5. CCL3 – Is upregulated in infiltrating immune cells after nerve injury [19]. Upregulated together with its receptor in the spinal dorsal horn after peripheral nerve injury and believed to be involved in the induction of neuropathic pain [20]. Involved in mechanical

allodynia [21]. Sensitizes TRPV1 [22]. Regulates macrophage response after nerve injury [15]. Increased in serum of ALS patients [23].

6. Nt3 – Neurotrophin. Regulates motor neuron phenotype [24]. Critical factor for hypersensitivity and pain following neonatal skin injury [25]. Suggested negative regulator of NGF and its pro-nociceptive activity [26]. Regulated in muscle tissue of ALS mouse model [27]. Reduced in ALS post mortem spinal cord [28].
7. Nt4 – Neurotrophin. Regulates motor neuron phenotype [24]. Necessary for the maintenance of spinal cord motor neurons [29]. Regulated in muscle tissue of ALS mouse model [27].
8. MMP2 – involved in the late phase development of neuropathic pain [30]. Elevated in spinal cord and skin of mouse ALS model [31]. Elevated in serum of ALS patients [32].
9. MMP9 – involved in the early phase development of neuropathic pain [30]. Elevated in spinal cord and skin of mouse ALS model [31]. Factor in selective neurodegeneration of fast motor neurons in ALS model [33].Elevated in serum of ALS patients and reduced in their CSF [32].
10. CX3CL1 –Involved in neuronal-glial communication, and spinal processing of neuropathic and inflammatory pain [12, 34-36]. Possible substrate for MMP9 for microglial activation in peripheral neuropathic pain models. [37]. Reduces inflammatory and neuropathic pain when knocked out [38]. Suggested involvement in inflammatory pain [39], radicular pain [40]. When released from therapeutic mesenchymal stem cells , it appears to have a calming effect on activated microglia [41] a function recently explored in a novel experimental ALS treatment [42].
11. nNOS – NMDAR activation leads induced nitric oxide (NO) production is implicated in inflammation and pain. [43]. nNOS-positive motor neurons are reduced in ALS mice [44].
12. Kallikrein 5 – The 15 kallikreins are relatively recently known and their role in the CNS is being mapped and they are considered promising drug targets [45, 46]
13. Kallikrein 14 – see above. Highly expressed in the CNS.
14. VEGF – inflammation. Recently found to be upregulated in ALS model spinal microglia phenotype [47]. Inhibiting VEGFR worsens peripheral neuronal damage [48] and VGFA and its receptor 2 are believed to be involved in neuropathic pain pathology [49]. Changing the balance between VEGF isoforms has been suggested as a new analgesic strategy [50].
15. IL4 – inflammation. Anti-inflammatory cytokine. Recombinant IL-4 could be a future treatment for neuropathic pain [51]. An ALS therapy optimizing model suggests injection of IL6 in order to generate high levels of neuroprotective IL4 as a promising option for ALS therapy [52].
16. IL6 – inflammation. Recently found to be downregulated spinal microglial phenotype in areas of neuronal destruction in ALS model [47]. Anti-IL6 treatment reduces peripheral nerve injury-induced mechanical allodynia [53]. Increased in the CSF of ALS patients compared to Alzheimer CSF [23]. Increased in serum of ALS patients [23]. Elevated in migraine patients serum during attacks [54]. An ALS therapy optimizing model suggests injection of IL6 in order to generate high levels of neuroprotective IL4 as a promising option for ALS therapy [52].
17. Cathepsin B - Microglial cathepsin B contributes to the initiation of peripheral inflammation-induced chronic pain.[55] Involved in the motor neuron degeneration of amyotrophic lateral sclerosis.[56] Repeatedly found to be altered in human ALS and ALS mouse model nervous tissue [57].

18. BDNF – Involved in the initiation and maintenance of chronic and/or neuropathic pain at the spinal, peripheral and central levels [58, 59]. Can also rescue spinal motor neurons from cell death [60] which is relevant for ALS. Upregulates cholinergic phenotype in motor neurons [24]. Recently found to be downregulated spinal microglial phenotype in areas of neuronal destruction in ALS model [47].
19. CF III – inflammation (innate immune response). Deposits are found in ALS model spinal cords [61]. Elevated in ALS patient CSF [62] and serum [62, 63]. It is suggested that local complement activation contributes to motor neuron death in ALS [64]. Abnormal activation seen in the development of neuropathic pain in a model [65].
20. Cathepsin S – cleaves CX3CL1 (for more see CX3CL1). Inhibiting it reduced pain like behaviors in an animal pain model [66].
21. CCL4 – CSF levels are positively correlated with ALSFRS-R score and negatively correlated with disease progression rate [13]. Increased in serum in from workers with musculoskeletal disorders [67].
22. CCL5 –Elevated in ALS CSF and serum suggestive of microglia induced recruitment of peripheral inflammatory cells to inflammatory sites in ALS patients [68]. Upregulated in mouse ALS model [14]. Elevated in painful intervertebral discs [69].
23. CXCL5 – Cytokine. Mediator of inflammatory pain [70, 71]. Increased in serum of ALS patients [23].
24. CD40 ligand - CD40 ligand-positive T lymphocytes have been found around damaged blood vessels in post mortem ALS patient spinal cord [72]. Anti- CD40L antibody treatment slowed weight loss, delayed paralysis and extended survival in an ALS mouse model [73]. Increased during high pain state in chronic fatigue patients [74, 75].
25. Cystatin B – Inhibits Cathepsin B (see cathepsin B).
26. E-selectin – A mutation in this gene has been identified as a genetic marker of sporadic ALS [76]. Elevated serum levels in ALS patients [77]. Elevated in lumbar discs of patients with radicular pain [78].
27. Cystatin C – A proteinase inhibitor and neuroprotective agent. Targeting it in motor neurons may provide a novel therapeutic strategy for ALS [79]. Although not a specific biomarker for ALS, serum levels may be an indicator of disease severity [80]. Elevated levels reported in CSF of patients with sciatic pain [81], but reported not to correlate with actual pain [82].
28. IL8 – Inflammation. Elevated in CSF of patients with fibromyalgia [83] and in serum of ALS patients [23].
29. ICAM-1 – Elevated in migraine patients serum during attacks [54]. Transcripts increased in spinal cord and dorsal root ganglia in neuropathic pain rat model [84]. Earliest upregulated gene in a mouse model of ALS [85].
30. P53 –Involved in ALS spinal cord motor neuron cell death [86]. Implicated in the pain-relieving effect of DHA treatment in neuropathic pain model [87].
31. IL1 beta – Inflammation. Possible substrate for MMP9 for microglial activation in peripheral neuropathic pain models. [37] Blocking IL1-signaling attenuates neuropathic pain like behavior [88]. Increased in serum of ALS patients [23]. Elevated in painful intervertebral discs [69]. Recruits immune cells after nerve injury [15].
32. EGF – Reduced in CSF of ALS patients compared to controls [89]. EGF treatment showed promise in an animal model of ALS [90]. Can block PGE2-induced sensitization in nociceptors [91].
33. Fas/TNFRSF6 – See TNF-alpha.
34. Follistatin – Direct activin A antagonist. Overexpressed in ALS skeletal muscle tissue [92].

35. HCC-4/CCL16 - Induces the expression of CCL2 [93] (see CCL2).
36. IL1 alpha – Inflammation. Increased in serum of ALS patients [23]. Displays anti-allodynic and anti-hyperalgesic activity in a rat neuropathic pain model [94].
37. IL10 – Inflammation (anti). A regulatory cytokine that supports an anti-inflammatory environment within the central nervous system [95]. Is required for CD4+ T cells to mediate neuroprotection in the CNS [96]. A promising experimental treatment in ALS mice appears to function through upregulation of IL-10 [97]. Provides analgesic effect in neuropathic pain model [98].
38. HGH – Human Growth Hormone (Somatotropin) Secretion is impaired in ALS patients [99]and has been shown to protect motor neurons in an ALS mouse model [100].
39. IL-17A – Inflammation. Contributes to neuroinflammation and neuropathic pain-like behavior in mouse model [101]. KO mice display attenuated pain phenotypes after nerve injury [102]. Increased in serum of ALS patients [12].
40. NGF-beta – Algesic. Important in the development and maintenance of nociceptors [1]. Central role in inflammation hyperalgesia. Anti-NGF is anti-hyperalgesic in humans [103, 104] and one of the most promising pain treatment developed in the last decade. Neurotrophic factors are involved in human sensory neuropathies [105]. Sensitizes nociceptors [7]. Neurotrophic factors are an important molecular category for new treatments in ALS and other neurodegenerative neurological diseases [106].
41. P-selectin – Inflammation. Contributes to neuropathic pain like behavior in pain model [107].
42. IL7 – Inflammation. Elevated in ALS patients [108].
43. Timp 4 – Acts on MMP-1, MMP-2, MMP-3, MMP-7 and MMP-9 (Uniprot).
44. Kallikrein 6 – Inflammation. Aggrevates glutamate neurotoxicity [109]. Believed to be involved in Alzheimer's disease pathology [110, 111].
45. GDF-15 – Neurotrophic factor suggested as a target for modulating inflammatory conditions [112].
46. TIMP-1 – Matrixmetallo proteinase inhibitor which can inhibit MMP9[113] and MMP2 (uniprot). Transcripts increased in spinal cord and dorsal root ganglia in neuropathic pain rat model [52]. Elevated in serum of ALS patients [32].
47. TNF-alpha – Inflammatory cytokine. Recently found to be downregulated spinal microglial phenotype in areas of neuronal destruction in ALS model [47]. Possible substrate for MMP9 for microglial activation in peripheral neuropathic pain models. [37] Implicated in neuropathic pain development and maintenance [114]. Increased in serum of ALS patients [23].

References

1. Lewin GR, Nykjaer A. Pro-neurotrophins, sortilin, and nociception. Eur J Neurosci. 2014;39(3):363-74. doi: 10.1111/ejn.12466. PubMed PMID: 24494677.
2. Vaegter CB, Jansen P, Fjorback AW, Glerup S, Skeldal S, Kjolby M, et al. Sortilin associates with Trk receptors to enhance anterograde transport and neurotrophin signaling. Nat Neurosci. 2011;14(1):54-61. doi: 10.1038/nn.2689. PubMed PMID: 21102451; PubMed Central PMCID: PMCPMC3808973.
3. Belzil VV, André-Guimont C, Atallah MR, Daoud H, Dupré N, Bouchard JP, et al. Analysis of the SORT1 gene in familial amyotrophic lateral sclerosis. Neurobiol Aging. 2012;33(8):1845.e7-9. doi: 10.1016/j.neurobiolaging.2012.01.011. PubMed PMID: 22361451.

4. Nykjaer A, Willnow TE. Sortilin: a receptor to regulate neuronal viability and function. Trends Neurosci. 2012;35(4):261-70. doi: 10.1016/j.tins.2012.01.003. PubMed PMID: 22341525.
5. Ricketts T, McGoldrick P, Fratta P, de Oliveira HM, Kent R, Phatak V, et al. A nonsense mutation in mouse Tardbp affects TDP43 alternative splicing activity and causes limb-clasping and body tone defects. PLoS One. 2014;9(1):e85962. doi: 10.1371/journal.pone.0085962. PubMed PMID: 24465814; PubMed Central PMCID: PMCPMC3897576.
6. Grundström E, Lindholm D, Johansson A, Blennow K, Askmark H. GDNF but not BDNF is increased in cerebrospinal fluid in amyotrophic lateral sclerosis. Neuroreport. 2000;11(8):1781-3. PubMed PMID: 10852244.
7. Malin SA, Molliver DC, Koerber HR, Cornuet P, Frye R, Albers KM, et al. Glial cell line-derived neurotrophic factor family members sensitize nociceptors in vitro and produce thermal hyperalgesia in vivo. J Neurosci. 2006;26(33):8588-99. doi: 10.1523/JNEUROSCI.1726-06.2006. PubMed PMID: 16914685.
8. Lundborg C, Hahn-Zoric M, Biber B, Hansson E. Glial cell line-derived neurotrophic factor is increased in cerebrospinal fluid but decreased in blood during long-term pain. J Neuroimmunol. 2010;220(1-2):108-13. doi: 10.1016/j.jneuroim.2010.01.007. PubMed PMID: 20129677.
9. Henderson CE, Phillips HS, Pollock RA, Davies AM, Lemeulle C, Armanini M, et al. GDNF: a potent survival factor for motoneurons present in peripheral nerve and muscle. Science. 1994;266(5187):1062-4. PubMed PMID: 7973664.
10. Albers KM, Zhang XL, Diges CM, Schwartz ES, Yang CI, Davis BM, et al. Artemin growth factor increases nicotinic cholinergic receptor subunit expression and activity in nociceptive sensory neurons. Mol Pain. 2014;10:31. doi: 10.1186/1744-8069-10-31. PubMed PMID: 24886596; PubMed Central PMCID: PMCPMC4036648.
11. Gardell LR, Wang R, Ehrenfels C, Ossipov MH, Rossomando AJ, Miller S, et al. Multiple actions of systemic artemin in experimental neuropathy. Nat Med. 2003;9(11):1383-9. doi: 10.1038/nm944. PubMed PMID: 14528299.
12. Gao YJ, Ji RR. Chemokines, neuronal-glial interactions, and central processing of neuropathic pain. Pharmacol Ther. 2010;126(1):56-68. doi: 10.1016/j.pharmthera.2010.01.002. PubMed PMID: 20117131; PubMed Central PMCID: PMCPMC2839017.
13. Tateishi T, Yamasaki R, Tanaka M, Matsushita T, Kikuchi H, Isobe N, et al. CSF chemokine alterations related to the clinical course of amyotrophic lateral sclerosis. J Neuroimmunol. 2010;222(1-2):76-81. doi: 10.1016/j.jneuroim.2010.03.004. PubMed PMID: 20381883.
14. Manzano R, Toivonen JM, Oliván S, Calvo AC, Moreno-Igoa M, Muñoz MJ, et al. Altered expression of myogenic regulatory factors in the mouse model of amyotrophic lateral sclerosis. Neurodegener Dis. 2011;8(5):386-96. doi: 10.1159/000324159. PubMed PMID: 21346327.
15. Perrin FE, Lacroix S, Avilés-Trigueros M, David S. Involvement of monocyte chemoattractant protein-1, macrophage inflammatory protein-1alpha and interleukin-1beta in Wallerian degeneration. Brain. 2005;128(Pt 4):854-66. doi: 10.1093/brain/awh407. PubMed PMID: 15689362.
16. Gao YJ, Zhang L, Samad OA, Suter MR, Yasuhiko K, Xu ZZ, et al. JNK-induced MCP-1 production in spinal cord astrocytes contributes to central sensitization and neuropathic pain. J Neurosci. 2009;29(13):4096-108. doi: 10.1523/JNEUROSCI.3623-08.2009. PubMed PMID: 19339605; PubMed Central PMCID: PMCPMC2682921.
17. Zhu X, Cao S, Zhu MD, Liu JQ, Chen JJ, Gao YJ. Contribution of Chemokine CCL2/CCR2 Signaling in the Dorsal Root Ganglion and Spinal Cord to the Maintenance of Neuropathic Pain in a Rat Model of Lumbar Disc Herniation. J Pain. 2014. doi: 10.1016/j.jpain.2014.01.492. PubMed PMID: 24462503.

18. Sato KL, Johanek LM, Sanada LS, Sluka KA. Spinal cord stimulation reduces mechanical hyperalgesia and glial cell activation in animals with neuropathic pain. Anesth Analg. 2014;118(2):464-72. doi: 10.1213/ANE.0000000000000047. PubMed PMID: 24361846.
19. Kiguchi N, Kobayashi Y, Saika F, Kishioka S. Epigenetic upregulation of CCL2 and CCL3 via histone modifications in infiltrating macrophages after peripheral nerve injury. Cytokine. 2013;64(3):666-72. doi: 10.1016/j.cyto.2013.09.019. PubMed PMID: 24135048.
20. Kiguchi N, Kobayashi Y, Maeda T, Saika F, Kishioka S. CC-chemokine MIP-1□ in the spinal cord contributes to nerve injury-induced neuropathic pain. Neurosci Lett. 2010;484(1):17-21. doi: 10.1016/j.neulet.2010.07.085. PubMed PMID: 20692319.
21. Ochi-ishi R, Nagata K, Inoue T, Tozaki-Saitoh H, Tsuda M, Inoue K. Involvement of the chemokine CCL3 and the purinoceptor P2X7 in the spinal cord in paclitaxel-induced mechanical allodynia. Mol Pain. 2014;10:53. doi: 10.1186/1744-8069-10-53. PubMed PMID: 25127716; PubMed Central PMCID: PMCPMC4141668.
22. Zhang N, Inan S, Cowan A, Sun R, Wang JM, Rogers TJ, et al. A proinflammatory chemokine, CCL3, sensitizes the heat- and capsaicin-gated ion channel TRPV1. Proc Natl Acad Sci U S A. 2005;102(12):4536-41. doi: 10.1073/pnas.0406030102. PubMed PMID: 15764707; PubMed Central PMCID: PMCPMC555471.
23. Mizwicki MT, Fiala M, Magpantay L, Aziz N, Sayre J, Liu G, et al. Tocilizumab attenuates inflammation in ALS patients through inhibition of IL6 receptor signaling. Am J Neurodegener Dis. 2012;1(3):305-15. PubMed PMID: 23383400; PubMed Central PMCID: PMCPMC3560466.
24. Wong V, Arriaga R, Ip NY, Lindsay RM. The neurotrophins BDNF, NT-3 and NT-4/5, but not NGF, up-regulate the cholinergic phenotype of developing motor neurons. Eur J Neurosci. 1993;5(5):466-74. PubMed PMID: 7505167.
25. Beggs S, Alvares D, Moss A, Currie G, Middleton J, Salter MW, et al. A role for NT-3 in the hyperinnervation of neonatally wounded skin. Pain. 2012;153(10):2133-9. doi: 10.1016/j.pain.2012.07.012. PubMed PMID: 22871470; PubMed Central PMCID: PMCPMC3657181.
26. Wilson-Gerwing TD, Dmyterko MV, Zochodne DW, Johnston JM, Verge VM. Neurotrophin-3 suppresses thermal hyperalgesia associated with neuropathic pain and attenuates transient receptor potential vanilloid receptor-1 expression in adult sensory neurons. J Neurosci. 2005;25(3):758-67. doi: 10.1523/JNEUROSCI.3909-04.2005. PubMed PMID: 15659614.
27. Harandi VM, Lindquist S, Kolan SS, Brännström T, Liu JX. Analysis of neurotrophic factors in limb and extraocular muscles of mouse model of amyotrophic lateral sclerosis. PLoS One. 2014;9(10):e109833. doi: 10.1371/journal.pone.0109833. PubMed PMID: 25334047; PubMed Central PMCID: PMCPMC4198138.
28. Duberley RM, Johnson IP, Anand P, Leigh PN, Cairns NJ. Neurotrophin-3-like immunoreactivity and Trk C expression in human spinal motoneurones in amyotrophic lateral sclerosis. J Neurol Sci. 1997;148(1):33-40. PubMed PMID: 9125388.
29. Stephens HE, Belliveau AC, Gupta JS, Mirkovic S, Kablar B. The role of neurotrophins in the maintenance of the spinal cord motor neurons and the dorsal root ganglia proprioceptive sensory neurons. Int J Dev Neurosci. 2005;23(7):613-20. doi: 10.1016/j.ijdevneu.2005.07.002. PubMed PMID: 16183241.
30. Kawasaki Y, Xu ZZ, Wang X, Park JY, Zhuang ZY, Tan PH, et al. Distinct roles of matrix metalloproteases in the early- and late-phase development of neuropathic pain. Nat Med. 2008;14(3):331-6. doi: 10.1038/nm1723. PubMed PMID: 18264108; PubMed Central PMCID: PMCPMC2279180.

31. Fang L, Teuchert M, Huber-Abel F, Schattauer D, Hendrich C, Dorst J, et al. MMP-2 and MMP-9 are elevated in spinal cord and skin in a mouse model of ALS. J Neurol Sci. 2010;294(1-2):51-6. doi: 10.1016/j.jns.2010.04.005. PubMed PMID: 20441996.
32. Niebroj-Dobosz I, Janik P, Soko□owska B, Kwiecinski H. Matrix metalloproteinases and their tissue inhibitors in serum and cerebrospinal fluid of patients with amyotrophic lateral sclerosis. Eur J Neurol. 2010;17(2):226-31. doi: 10.1111/j.1468-1331.2009.02775.x. PubMed PMID: 19796283.
33. Kaplan A, Spiller KJ, Towne C, Kanning KC, Choe GT, Geber A, et al. Neuronal matrix metalloproteinase-9 is a determinant of selective neurodegeneration. Neuron. 2014;81(2):333-48. doi: 10.1016/j.neuron.2013.12.009. PubMed PMID: 24462097.
34. Clark AK, Staniland AA, Malcangio M. Fractalkine/CX3CR1 signalling in chronic pain and inflammation. Curr Pharm Biotechnol. 2011;12(10):1707-14. PubMed PMID: 21466443.
35. Sun JL, Xiao C, Lu B, Zhang J, Yuan XZ, Chen W, et al. CX3CL1/CX3CR1 regulates nerve injury-induced pain hypersensitivity through the ERK5 signaling pathway. J Neurosci Res. 2013;91(4):545-53. doi: 10.1002/jnr.23168. PubMed PMID: 23361876.
36. Kiyomoto M, Shinoda M, Okada-Ogawa A, Noma N, Shibuta K, Tsuboi Y, et al. Fractalkine signaling in microglia contributes to ectopic orofacial pain following trapezius muscle inflammation. J Neurosci. 2013;33(18):7667-80. doi: 10.1523/JNEUROSCI.4968-12.2013. PubMed PMID: 23637160.
37. Suter MR, Wen YR, Decosterd I, Ji RR. Do glial cells control pain? Neuron Glia Biol. 2007;3(3):255-68. doi: 10.1017/S1740925X08000100. PubMed PMID: 18504511; PubMed Central PMCID: PMCPMC2394739.
38. Staniland AA, Clark AK, Wodarski R, Sasso O, Maione F, D'Acquisto F, et al. Reduced inflammatory and neuropathic pain and decreased spinal microglial response in fractalkine receptor (CX3CR1) knockout mice. J Neurochem. 2010;114(4):1143-57. doi: 10.1111/j.1471-4159.2010.06837.x. PubMed PMID: 20524966.
39. Willemen HL, Eijkelkamp N, Wang H, Dantzer R, Dorn GW, Kelley KW, et al. Microglial/macrophage GRK2 determines duration of peripheral IL-1beta-induced hyperalgesia: contribution of spinal cord CX3CR1, p38 and IL-1 signaling. Pain. 2010;150(3):550-60. doi: 10.1016/j.pain.2010.06.015. PubMed PMID: 20609517; PubMed Central PMCID: PMCPMC3099597.
40. Park HW, Ahn SH, Kim SJ, Seo JM, Cho YW, Jang SH, et al. Changes in spinal cord expression of fractalkine and its receptor in a rat model of disc herniation by autologous nucleus pulposus. Spine (Phila Pa 1976). 2011;36(12):E753-60. doi: 10.1097/BRS.0b013e3181ef610b. PubMed PMID: 21224760.
41. Giunti D, Parodi B, Usai C, Vergani L, Casazza S, Bruzzone S, et al. Mesenchymal stem cells shape microglia effector functions through the release of CX3CL1. Stem Cells. 2012;30(9):2044-53. doi: 10.1002/stem.1174. PubMed PMID: 22821677.
42. Sun H, Bénardais K, Stanslowsky N, Thau-Habermann N, Hensel N, Huang D, et al. Therapeutic potential of mesenchymal stromal cells and MSC conditioned medium in Amyotrophic Lateral Sclerosis (ALS)--in vitro evidence from primary motor neuron cultures, NSC-34 cells, astrocytes and microglia. PLoS One. 2013;8(9):e72926. doi: 10.1371/journal.pone.0072926. PubMed PMID: 24069165; PubMed Central PMCID: PMCPMC3771979.
43. Miclescu A, Gordh T. Nitric oxide and pain: 'Something old, something new'. Acta Anaesthesiol Scand. 2009;53(9):1107-20. doi: 10.1111/j.1399-6576.2009.02054.x. PubMed PMID: 19702699.
44. Lee J, Ryu H, Kowall NW. Differential regulation of neuronal and inducible nitric oxide synthase (NOS) in the spinal cord of mutant SOD1 (G93A) ALS mice. Biochem Biophys Res

Commun. 2009;387(1):202-6. doi: 10.1016/j.bbrc.2009.07.007. PubMed PMID: 19580782; PubMed Central PMCID: PMCPMC2742676.
45. Yousef GM, Kishi T, Diamandis EP. Role of kallikrein enzymes in the central nervous system. Clin Chim Acta. 2003;329(1-2):1-8. PubMed PMID: 12589961.
46. Prassas I, Eissa A, Poda G, Diamandis EP. Unleashing the therapeutic potential of human kallikrein-related serine proteases. Nat Rev Drug Discov. 2015;14(3):183-202. doi: 10.1038/nrd4534. PubMed PMID: 25698643.
47. Nikodemova M, Small AL, Smith SM, Mitchell GS, Watters JJ. Spinal but not cortical microglia acquire an atypical phenotype with high VEGF, galectin-3 and osteopontin, and blunted inflammatory responses in ALS rats. Neurobiol Dis. 2014;69:43-53. doi: 10.1016/j.nbd.2013.11.009. PubMed PMID: 24269728; PubMed Central PMCID: PMCPMC4079765.
48. Beazley-Long N, Hua J, Jehle T, Hulse RP, Dersch R, Lehrling C, et al. VEGF-A165b is an endogenous neuroprotective splice isoform of vascular endothelial growth factor A in vivo and in vitro. Am J Pathol. 2013;183(3):918-29. doi: 10.1016/j.ajpath.2013.05.031. PubMed PMID: 23838428; PubMed Central PMCID: PMCPMC3763768.
49. Lin J, Li G, Den X, Xu C, Liu S, Gao Y, et al. VEGF and its receptor-2 involved in neuropathic pain transmission mediated by $P2X_2(/)_3$ receptor of primary sensory neurons. Brain Res Bull. 2010;83(5):284-91. doi: 10.1016/j.brainresbull.2010.08.002. PubMed PMID: 20705122.
50. Hulse RP, Beazley-Long N, Hua J, Kennedy H, Prager J, Bevan H, et al. Regulation of alternative VEGF-A mRNA splicing is a therapeutic target for analgesia. Neurobiol Dis. 2014;71:245-59. doi: 10.1016/j.nbd.2014.08.012. PubMed PMID: 25151644; PubMed Central PMCID: PMCPMC4194316.
51. Austin PJ, Moalem-Taylor G. The neuro-immune balance in neuropathic pain: involvement of inflammatory immune cells, immune-like glial cells and cytokines. J Neuroimmunol. 2010;229(1-2):26-50. doi: 10.1016/j.jneuroim.2010.08.013. PubMed PMID: 20870295.
52. Shao H, He Y, Li KC, Zhou X. A system mathematical model of a cell-cell communication network in amyotrophic lateral sclerosis. Mol Biosyst. 2013;9(3):398-406. doi: 10.1039/c2mb25370d. PubMed PMID: 23287963; PubMed Central PMCID: PMCPMC3752652.
53. Arruda JL, Sweitzer S, Rutkowski MD, DeLeo JA. Intrathecal anti-IL-6 antibody and IgG attenuates peripheral nerve injury-induced mechanical allodynia in the rat: possible immune modulation in neuropathic pain. Brain Res. 2000;879(1-2):216-25. PubMed PMID: 11011025.
54. Wang F, He Q, Ren Z, Li F, Chen W, Lin X, et al. Association of serum levels of intercellular adhesion molecule-1 and interleukin-6 with migraine. Neurol Sci. 2015;36(4):535-40. doi: 10.1007/s10072-014-2010-3. PubMed PMID: 25417066.
55. Sun L, Wu Z, Hayashi Y, Peters C, Tsuda M, Inoue K, et al. Microglial cathepsin B contributes to the initiation of peripheral inflammation-induced chronic pain. J Neurosci. 2012;32(33):11330-42. doi: 10.1523/JNEUROSCI.0677-12.2012. PubMed PMID: 22895716.
56. Kikuchi H, Yamada T, Furuya H, Doh-ura K, Ohyagi Y, Iwaki T, et al. Involvement of cathepsin B in the motor neuron degeneration of amyotrophic lateral sclerosis. Acta Neuropathol. 2003;105(5):462-8. doi: 10.1007/s00401-002-0667-9. PubMed PMID: 12677446.
57. Saris CG, Groen EJ, Koekkoek JA, Veldink JH, van den Berg LH. Meta-analysis of gene expression profiling in amyotrophic lateral sclerosis: a comparison between transgenic mouse models and human patients. Amyotroph Lateral Scler Frontotemporal Degener. 2013;14(3):177-89. doi: 10.3109/21678421.2012.729842. PubMed PMID: 23286751.
58. Smith PA. BDNF: no gain without pain? Neuroscience. 2014;283:107-23. doi: 10.1016/j.neuroscience.2014.05.044. PubMed PMID: 24887639.

59. Obata K, Yamanaka H, Dai Y, Tachibana T, Fukuoka T, Tokunaga A, et al. Differential activation of extracellular signal-regulated protein kinase in primary afferent neurons regulates brain-derived neurotrophic factor expression after peripheral inflammation and nerve injury. J Neurosci. 2003;23(10):4117-26. PubMed PMID: 12764099.
60. Yan Q, Elliott J, Snider WD. Brain-derived neurotrophic factor rescues spinal motor neurons from axotomy-induced cell death. Nature. 1992;360(6406):753-5. doi: 10.1038/360753a0. PubMed PMID: 1281520.
61. Woodruff TM, Costantini KJ, Crane JW, Atkin JD, Monk PN, Taylor SM, et al. The complement factor C5a contributes to pathology in a rat model of amyotrophic lateral sclerosis. J Immunol. 2008;181(12):8727-34. PubMed PMID: 19050293.
62. Annunziata P, Volpi N. High levels of C3c in the cerebrospinal fluid from amyotrophic lateral sclerosis patients. Acta Neurol Scand. 1985;72(1):61-4. PubMed PMID: 4050318.
63. Goldknopf IL, Sheta EA, Bryson J, Folsom B, Wilson C, Duty J, et al. Complement C3c and related protein biomarkers in amyotrophic lateral sclerosis and Parkinson's disease. Biochem Biophys Res Commun. 2006;342(4):1034-9. doi: 10.1016/j.bbrc.2006.02.051. PubMed PMID: 16516157.
64. Lee JD, Kamaruzaman NA, Fung JN, Taylor SM, Turner BJ, Atkin JD, et al. Dysregulation of the complement cascade in the hSOD1G93A transgenic mouse model of amyotrophic lateral sclerosis. J Neuroinflammation. 2013;10:119. doi: 10.1186/1742-2094-10-119. PubMed PMID: 24067070; PubMed Central PMCID: PMCPMC3850877.
65. Nie F, Wang J, Su D, Shi Y, Chen J, Wang H, et al. Abnormal activation of complement C3 in the spinal dorsal horn is closely associated with progression of neuropathic pain. Int J Mol Med. 2013;31(6):1333-42. doi: 10.3892/ijmm.2013.1344. PubMed PMID: 23588254.
66. Clark AK, Grist J, Al-Kashi A, Perretti M, Malcangio M. Spinal cathepsin S and fractalkine contribute to chronic pain in the collagen-induced arthritis model. Arthritis Rheum. 2012;64(6):2038-47. doi: 10.1002/art.34351. PubMed PMID: 22213084.
67. Matute Wilander A, Kåredal M, Axmon A, Nordander C. Inflammatory biomarkers in serum in subjects with and without work related neck/shoulder complaints. BMC Musculoskelet Disord. 2014;15:103. doi: 10.1186/1471-2474-15-103. PubMed PMID: 24669872; PubMed Central PMCID: PMCPMC3973377.
68. Rentzos M, Nikolaou C, Rombos A, Boufidou F, Zoga M, Dimitrakopoulos A, et al. RANTES levels are elevated in serum and cerebrospinal fluid in patients with amyotrophic lateral sclerosis. Amyotroph Lateral Scler. 2007;8(5):283-7. doi: 10.1080/17482960701419232. PubMed PMID: 17852013.
69. Kepler CK, Markova DZ, Dibra F, Yadla S, Vaccaro AR, Risbud MV, et al. Expression and relationship of proinflammatory chemokine RANTES/CCL5 and cytokine IL-1[illegible] in painful human intervertebral discs. Spine (Phila Pa 1976). 2013;38(11):873-80. doi: 10.1097/BRS.0b013e318285ae08. PubMed PMID: 23660804.
70. Dawes JM, Calvo M, Perkins JR, Paterson KJ, Kiesewetter H, Hobbs C, et al. CXCL5 mediates UVB irradiation-induced pain. Sci Transl Med. 2011;3(90):90ra60. doi: 10.1126/scitranslmed.3002193. PubMed PMID: 21734176; PubMed Central PMCID: PMCPMC3232447.
71. Dawes JM, Antunes-Martins A, Perkins JR, Paterson KJ, Sisignano M, Schmid R, et al. Genome-wide transcriptional profiling of skin and dorsal root ganglia after ultraviolet-B-induced inflammation. PLoS One. 2014;9(4):e93338. doi: 10.1371/journal.pone.0093338. PubMed PMID: 24732968; PubMed Central PMCID: PMCPMC3986071.
72. Graves MC, Fiala M, Dinglasan LA, Liu NQ, Sayre J, Chiappelli F, et al. Inflammation in amyotrophic lateral sclerosis spinal cord and brain is mediated by activated macrophages, mast

cells and T cells. Amyotroph Lateral Scler Other Motor Neuron Disord. 2004;5(4):213-9. PubMed PMID: 15799549.
73. Lincecum JM, Vieira FG, Wang MZ, Thompson K, De Zutter GS, Kidd J, et al. From transcriptome analysis to therapeutic anti-CD40L treatment in the SOD1 model of amyotrophic lateral sclerosis. Nat Genet. 2010;42(5):392-9. doi: 10.1038/ng.557. PubMed PMID: 20348957.
74. White AT, Light AR, Hughen RW, Bateman L, Martins TB, Hill HR, et al. Severity of symptom flare after moderate exercise is linked to cytokine activity in chronic fatigue syndrome. Psychophysiology. 2010;47(4):615-24. doi: 10.1111/j.1469-8986.2010.00978.x. PubMed PMID: 20230500; PubMed Central PMCID: PMCPMC4378647.
75. DeVon HA, Piano MR, Rosenfeld AG, Hoppensteadt DA. The association of pain with protein inflammatory biomarkers: a review of the literature. Nurs Res. 2014;63(1):51-62. doi: 10.1097/NNR.0000000000000013. PubMed PMID: 24335913.
76. Penco S, Buscema M, Patrosso MC, Marocchi A, Grossi E. New application of intelligent agents in sporadic amyotrophic lateral sclerosis identifies unexpected specific genetic background. BMC Bioinformatics. 2008;9:254. doi: 10.1186/1471-2105-9-254. PubMed PMID: 18513389; PubMed Central PMCID: PMCPMC2443147.
77. Ikeda J, Kohriyama T, Nakamura S. Elevation of serum soluble E-selectin and antisulfoglucuronyl paragloboside antibodies in amyotrophic lateral sclerosis. Eur J Neurol. 2000;7(5):541-7. PubMed PMID: 11054140.
78. Tufan K, Sen O, Cekinmez M, Bolat FA, Alkan O, Sarica FB, et al. Comparison of E-selectin and the other inflammatory markers in lumbar disc herniation: a new promising therapeutical window for radicular pain. J Spinal Disord Tech. 2012;25(8):443-6. doi: 10.1097/BSD.0b013e318238e2db. PubMed PMID: 22015628.
79. Watanabe S, Hayakawa T, Wakasugi K, Yamanaka K. Cystatin C protects neuronal cells against mutant copper-zinc superoxide dismutase-mediated toxicity. Cell Death Dis. 2014;5:e1497. doi: 10.1038/cddis.2014.459. PubMed PMID: 25356866; PubMed Central PMCID: PMCPMC4237269.
80. Ren Y, Zhu W, Cui F, Yang F, Chen Z, Ling L, et al. Measurement of cystatin C levels in the cerebrospinal fluid of patients with amyotrophic lateral sclerosis. Int J Clin Exp Pathol. 2015;8(5):5419-26. PubMed PMID: 26191245; PubMed Central PMCID: PMCPMC4503116.
81. Liu X, Zeng B, Xu J. Alteration of cystatin C in cerebrospinal fluid of patients with sciatica revealed by a proteomical approach. Saudi Med J. 2005;26(11):1699-704. PubMed PMID: 16311651.
82. Eisenach JC, Thomas JA, Rauck RL, Curry R, Li X. Cystatin C in cerebrospinal fluid is not a diagnostic test for pain in humans. Pain. 2004;107(3):207-12. PubMed PMID: 14736582.
83. Kosek E, Altawil R, Kadetoff D, Finn A, Westman M, Le Maître E, et al. Evidence of different mediators of central inflammation in dysfunctional and inflammatory pain--interleukin-8 in fibromyalgia and interleukin-1 [illegible] in rheumatoid arthritis. J Neuroimmunol. 2015;280:49-55. doi: 10.1016/j.jneuroim.2015.02.002. PubMed PMID: 25773155; PubMed Central PMCID: PMCPMC4372266.
84. Rodriguez Parkitna J, Korostynski M, Kaminska-Chowaniec D, Obara I, Mika J, Przewlocka B, et al. Comparison of gene expression profiles in neuropathic and inflammatory pain. J Physiol Pharmacol. 2006;57(3):401-14. PubMed PMID: 17033093.
85. Alexianu ME, Kozovska M, Appel SH. Immune reactivity in a mouse model of familial ALS correlates with disease progression. Neurology. 2001;57(7):1282-9. PubMed PMID: 11591849.
86. Ranganathan S, Bowser R. p53 and Cell Cycle Proteins Participate in Spinal Motor Neuron Cell Death in ALS. Open Pathol J. 2010;4:11-22. doi: 10.2174/1874375701004010011. PubMed PMID: 21572928; PubMed Central PMCID: PMCPMC3092395.

87. Manzhulo IV, Ogurtsova OS, Lamash NE, Latyshev NA, Kasyanov SP, Dyuizen IV. Analgetic effect of docosahexaenoic acid is mediated by modulating the microglia activity in the dorsal root ganglia in a rat model of neuropathic pain. Acta Histochem. 2015;117(7):659-66. doi: 10.1016/j.acthis.2015.07.001. PubMed PMID: 26182833.
88. Wolf G, Gabay E, Tal M, Yirmiya R, Shavit Y. Genetic impairment of interleukin-1 signaling attenuates neuropathic pain, autotomy, and spontaneous ectopic neuronal activity, following nerve injury in mice. Pain. 2006;120(3):315-24. doi: 10.1016/j.pain.2005.11.011. PubMed PMID: 16426759.
89. Cie□lak D, Szulc-Kuberska J, Stepie□ H, Klimek A. Epidermal growth factor in human cerebrospinal fluid: reduced levels in amyotrophic lateral sclerosis. J Neurol. 1986;233(6):376-7. PubMed PMID: 3492593.
90. Del Barco DG, Pérez-Saad H, Rodríguez V, Marín J, Falcón V, Martín J, et al. Therapeutic effect of the combined use of growth hormone releasing peptide-6 and epidermal growth factor in an axonopathy model. Neurotox Res. 2011;19(1):195-209. doi: 10.1007/s12640-010-9160-8. PubMed PMID: 20169434.
91. Andres C, Meyer S, Dina OA, Levine JD, Hucho T. Quantitative automated microscopy (QuAM) elucidates growth factor specific signalling in pain sensitization. Mol Pain. 2010;6:98. doi: 10.1186/1744-8069-6-98. PubMed PMID: 21187008; PubMed Central PMCID: PMCPMC3023724.
92. Shtilbans A, Choi SG, Fowkes ME, Khitrov G, Shahbazi M, Ting J, et al. Differential gene expression in patients with amyotrophic lateral sclerosis. Amyotroph Lateral Scler. 2011;12(4):250-6. doi: 10.3109/17482968.2011.560946. PubMed PMID: 21375368.
93. Musso T, Cappello P, Stornello S, Ravarino D, Caorsi C, Otero K, et al. IL-10 enhances CCL2 release and chemotaxis induced by CCL16 in human monocytes. Int J Immunopathol Pharmacol. 2005;18(2):339-49. PubMed PMID: 15888256.
94. Mika J, Korostynski M, Kaminska D, Wawrzczak-Bargiela A, Osikowicz M, Makuch W, et al. Interleukin-1 alpha has antiallodynic and antihyperalgesic activities in a rat neuropathic pain model. Pain. 2008;138(3):587-97. doi: 10.1016/j.pain.2008.02.015. PubMed PMID: 18374486.
95. Strle K, Zhou JH, Shen WH, Broussard SR, Johnson RW, Freund GG, et al. Interleukin-10 in the brain. Crit Rev Immunol. 2001;21(5):427-49. PubMed PMID: 11942558.
96. Xin J, Wainwright DA, Mesnard NA, Serpe CJ, Sanders VM, Jones KJ. IL-10 within the CNS is necessary for CD4+ T cells to mediate neuroprotection. Brain Behav Immun. 2011;25(5):820-9. doi: 10.1016/j.bbi.2010.08.004. PubMed PMID: 20723599; PubMed Central PMCID: PMCPMC3021103.
97. Goursaud S, Schäfer S, Dumont AO, Vergouts M, Gallo A, Desmet N, et al. The anti-inflammatory peptide stearyl-norleucine-VIP delays disease onset and extends survival in a rat model of inherited amyotrophic lateral sclerosis. Exp Neurol. 2015;263:91-101. doi: 10.1016/j.expneurol.2014.09.022. PubMed PMID: 25311268.
98. Milligan ED, Sloane EM, Langer SJ, Hughes TS, Jekich BM, Frank MG, et al. Repeated intrathecal injections of plasmid DNA encoding interleukin-10 produce prolonged reversal of neuropathic pain. Pain. 2006;126(1-3):294-308. doi: 10.1016/j.pain.2006.07.009. PubMed PMID: 16949747.
99. Morselli LL, Bongioanni P, Genovesi M, Licitra R, Rossi B, Murri L, et al. Growth hormone secretion is impaired in amyotrophic lateral sclerosis. Clin Endocrinol (Oxf). 2006;65(3):385-8. doi: 10.1111/j.1365-2265.2006.02609.x. PubMed PMID: 16918961.
100. Chung JY, Kim HJ, Kim M. The protective effect of growth hormone on Cu/Zn superoxide dismutase-mutant motor neurons. BMC Neurosci. 2015;16:1. doi: 10.1186/s12868-015-0140-z. PubMed PMID: 25655275; PubMed Central PMCID: PMCPMC4326297.

101. Kim CF, Moalem-Taylor G. Interleukin-17 contributes to neuroinflammation and neuropathic pain following peripheral nerve injury in mice. J Pain. 2011;12(3):370-83. doi: 10.1016/j.jpain.2010.08.003. PubMed PMID: 20889388.
102. Day YJ, Liou JT, Lee CM, Lin YC, Mao CC, Chou AH, et al. Lack of interleukin-17 leads to a modulated micro-environment and amelioration of mechanical hypersensitivity after peripheral nerve injury in mice. Pain. 2014;155(7):1293-302. doi: 10.1016/j.pain.2014.04.004. PubMed PMID: 24721689.
103. Jimenez-Andrade JM, Ghilardi JR, Castañeda-Corral G, Kuskowski MA, Mantyh PW. Preventive or late administration of anti-NGF therapy attenuates tumor-induced nerve sprouting, neuroma formation, and cancer pain. Pain. 2011;152(11):2564-74. doi: 10.1016/j.pain.2011.07.020. PubMed PMID: 21907491; PubMed Central PMCID: PMCPMC3199350.
104. Mantyh WG, Jimenez-Andrade JM, Stake JI, Bloom AP, Kaczmarska MJ, Taylor RN, et al. Blockade of nerve sprouting and neuroma formation markedly attenuates the development of late stage cancer pain. Neuroscience. 2010;171(2):588-98. doi: 10.1016/j.neuroscience.2010.08.056. PubMed PMID: 20851743; PubMed Central PMCID: PMCPMC2992976.
105. Anand P. Neurotrophic factors and their receptors in human sensory neuropathies. Prog Brain Res. 2004;146:477-92. doi: 10.1016/S0079-6123(03)46030-5. PubMed PMID: 14699981.
106. Schulte-Herbrüggen O, Braun A, Rochlitzer S, Jockers-Scherübl MC, Hellweg R. Neurotrophic factors--a tool for therapeutic strategies in neurological, neuropsychiatric and neuroimmunological diseases? Curr Med Chem. 2007;14(22):2318-29. PubMed PMID: 17896980.
107. Liou JT, Lee CM, Lin YC, Chen CY, Liao CC, Lee HC, et al. P-selectin is required for neutrophils and macrophage infiltration into injured site and contributes to generation of behavioral hypersensitivity following peripheral nerve injury in mice. Pain. 2013;154(10):2150-9. doi: 10.1016/j.pain.2013.06.042. PubMed PMID: 23831400.
108. Furukawa T, Matsui N, Fujita K, Nodera H, Shimizu F, Miyamoto K, et al. CSF cytokine profile distinguishes multifocal motor neuropathy from progressive muscular atrophy. Neurol Neuroimmunol Neuroinflamm. 2015;2(5):e138. doi: 10.1212/NXI.0000000000000138. PubMed PMID: 26280014; PubMed Central PMCID: PMCPMC4529282.
109. Yoon H, Radulovic M, Wu J, Blaber SI, Blaber M, Fehlings MG, et al. Kallikrein 6 signals through PAR1 and PAR2 to promote neuron injury and exacerbate glutamate neurotoxicity. J Neurochem. 2013;127(2):283-98. doi: 10.1111/jnc.12293. PubMed PMID: 23647384; PubMed Central PMCID: PMCPMC4097186.
110. Ogawa K, Yamada T, Tsujioka Y, Taguchi J, Takahashi M, Tsuboi Y, et al. Localization of a novel type trypsin-like serine protease, neurosin, in brain tissues of Alzheimer's disease and Parkinson's disease. Psychiatry Clin Neurosci. 2000;54(4):419-26. doi: 10.1046/j.1440-1819.2000.00731.x. PubMed PMID: 10997858.
111. Diamandis EP, Yousef GM, Petraki C, Soosaipillai AR. Human kallikrein 6 as a biomarker of alzheimer's disease. Clin Biochem. 2000;33(8):663-7. PubMed PMID: 11166014.
112. Yi MH, Zhang E, Baek H, Kim S, Shin N, Kang JW, et al. Growth Differentiation Factor 15 Expression in Astrocytes After Excitotoxic Lesion in the Mouse Hippocampus. Exp Neurobiol. 2015;24(2):133-8. doi: 10.5607/en.2015.24.2.133. PubMed PMID: 26113792; PubMed Central PMCID: PMCPMC4479809.
113. Huang B, Zhao X, Zheng LB, Zhang L, Ni B, Wang YW. Different expression of tissue inhibitor of metalloproteinase family members in rat dorsal root ganglia and their changes after peripheral nerve injury. Neuroscience. 2011;193:421-8. doi: 10.1016/j.neuroscience.2011.07.031. PubMed PMID: 21782897.

114. Schäfers M, Lee DH, Brors D, Yaksh TL, Sorkin LS. Increased sensitivity of injured and adjacent uninjured rat primary sensory neurons to exogenous tumor necrosis factor-alpha after spinal nerve ligation. J Neurosci. 2003;23(7):3028-38. PubMed PMID: 12684490.
