# Supplementary material for: A Multiplex Protein Panel Applied to Cerebrospinal Fluid Reveals Three New Biomarker Candidates in ALS but None in Neuropathic Pain Patients
Source: PLoS One. 2016 Feb 25;11(2):e0149821. doi: 10.1371/journal.pone.0149821 (PMC4767403; doi:10.1371/journal.pone.0149821)
Supplement: S2 Appendix — (PDF) [file pone.0149821.s002.pdf]

Appendix 2

Outlier detection. Before fitting a five parameter log-logistic function to the standard curve measurement, outliers were detected and removed. Outliers at each concentration were detected using Grubb's test applying the 0.05 significance level. Iterative outlier detection was performed on the residuals. Outliers that were not detected in this global procedure were removed from the set of outliers but no further outliers were added. The background was defined as all samples at concentrations that did not differ significantly (at the 0.05 level) from the negative control (blank) samples according to a t-test. Only outliers that were identified in the iterative Grubb's test on the new blanks were kept.

Multivariate modeling. The multivariate modeling procedure is performed as follows:

1. Divide data into training and test data sets. 20% of the samples are randomly selected as test data and the remaining samples are used for training. The random selection is done in a balanced way so that the proportions of patients and controls are (approximately) the same in the test and training sets.

2. On the training data, compute Mann-Whitney U test statistics for all variables (biomarkers) and sort them according the statistic (highest (i.e. lowest p-value) first).

3. Select the k best variables, where k is the number between 1 and 10 that maximize the average accuracy in an internal 5-fold cross validaton.

4. Build a randomForest model using the training data including only the selected k variables.

5. Use the model to predict class identity (patient or control) for the test examples.

6. Evaluate the performance by computing the accuracy for the prediction on the test examples.

7. Repeat 1-6 100 times.

8. Compute the average accuracy over all 100 holdouts.
9. As a measure of variable importance, compute for each variable the fraction of holdouts in which it was selected (step 3).
