# Supplementary figures and images for: A Multiplex Protein Panel Applied to Cerebrospinal Fluid Reveals Three New Biomarker Candidates in ALS but None in Neuropathic Pain Patients
Source: PLoS One. 2016 Feb 25;11(2):e0149821. doi: 10.1371/journal.pone.0149821 (PMC4767403; doi:10.1371/journal.pone.0149821)

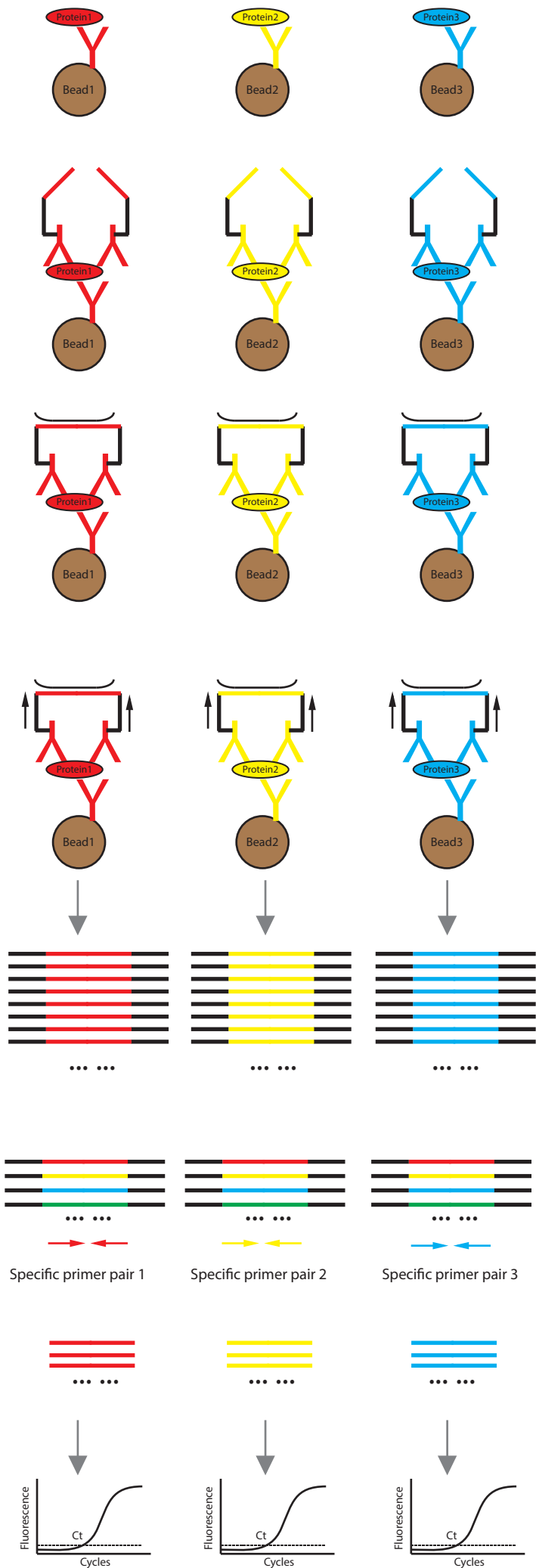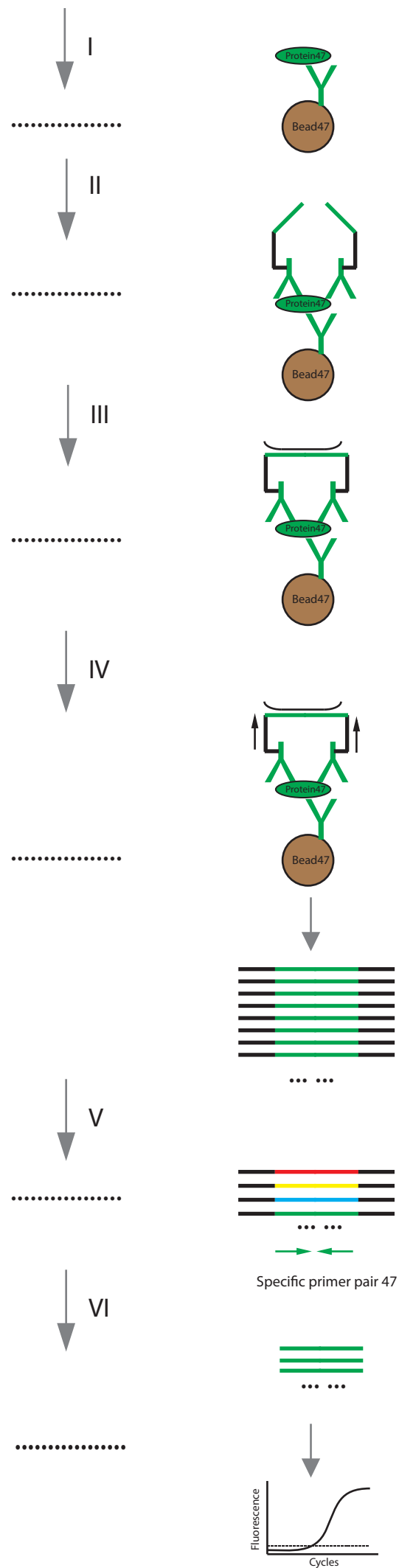

Supplement: S1 Fig — I) Samples are incubated with a mixture of magnetic beads, each equipped with one immobilized capture antibody against one of the 47 target proteins. II) After washing, the mixture is incubated with 47 pairs of PLA probes (antibodies with conjugated DNA strands). III) After a second wash, a ligation reaction is performed in the presence of a connector DNA oligonucleotide to allow enzymatic joining of the two PLA probes to form a new amplifiable DNA molecule. IV) After a third washing step, PCR is performed using a pair of universal PCR primers. V) The PCR products are then diluted in a new PCR mix and aliquoted to 47 wells in a 384-well plate, each well pre-spotted with a specific pair of PCR primers for each investigated protein, and VI) real time PCR is performed and the Ct values are collected for data analysis. (PDF) [file pone.0149821.s003.pdf]

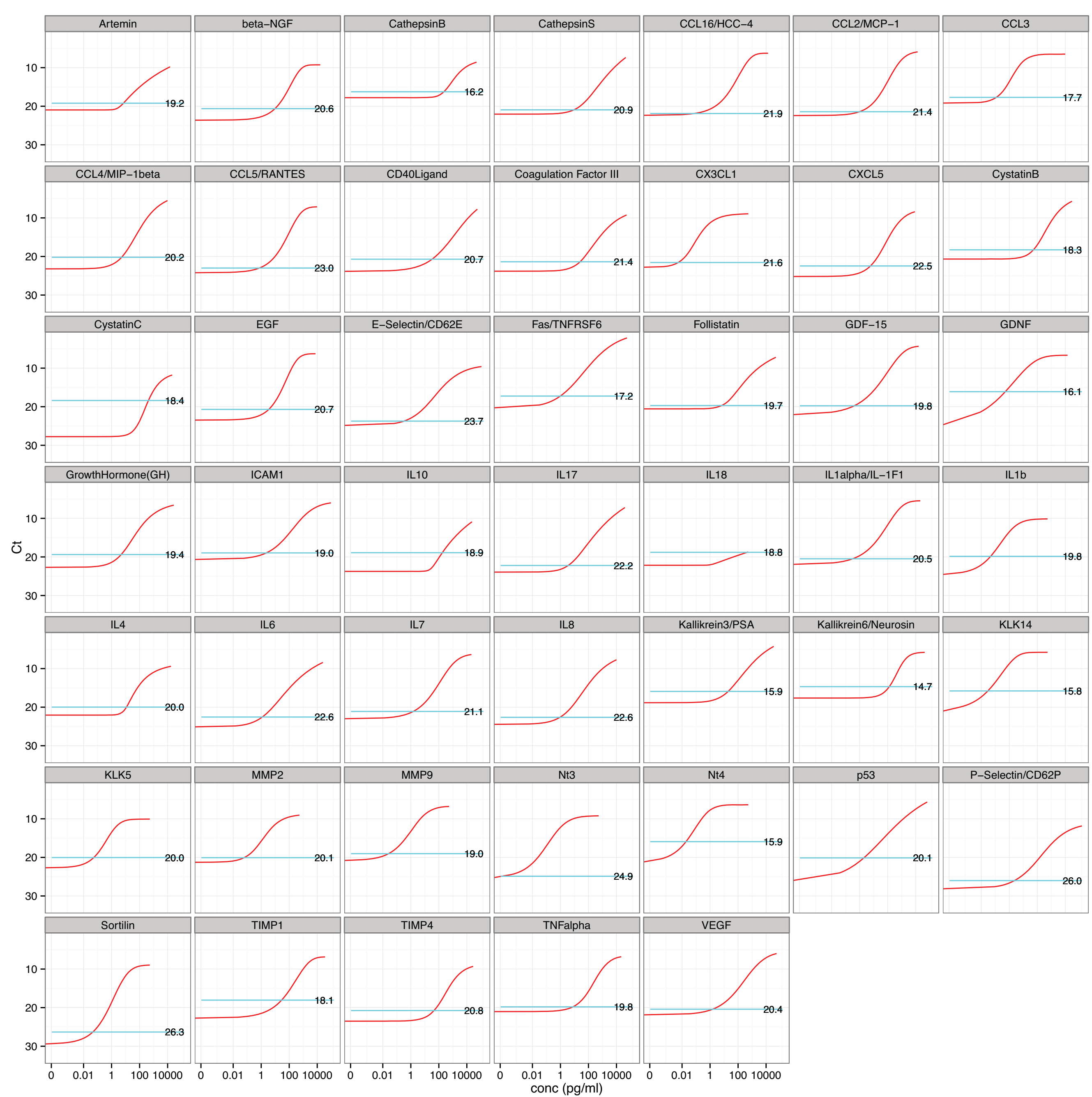

Supplement: S2 Fig — The fitted standard curve is shown in red and the values used to fit the standard curve are shown as black circles, the filled black circles are the measurements used as blanks. The LODs are shown as a cyan line and the LOD Ct-value is printed in the plots. The x-axis is shown in log-scale. Values along the x and y axes are indicated at the bottom and far left of the figure, respectively. (PDF) [file pone.0149821.s004.pdf]
