# Supplementary material for: A Multiplex Protein Panel Applied to Cerebrospinal Fluid Reveals Three New Biomarker Candidates in ALS but None in Neuropathic Pain Patients
Source: PLoS One. 2016 Feb 25;11(2):e0149821. doi: 10.1371/journal.pone.0149821 (PMC4767403; doi:10.1371/journal.pone.0149821)
Supplement: S1 Table — (PDF) [file pone.0149821.s005.pdf]

S1 Table. Characteristics of clinical samples

Controls	Age	Gender
1	60	Femal
2	84	Femal
3	82	Femal
4	76	Femal
5	71	Femal
6	68	Femal
7	64	Femal
8	58	Femal
9	55	Femal
10	51	Femal
11	66	Male
12	66	Male
13	66	Male
14	65	Male
15	65	Male
16	65	Male
17	65	Male
18	65	Male
19	65	Male
20	65	Male
**ALS patients**		
1	47	Femal
2	60	Femal
3	63	Femal
4	67	Femal
5	54	Femal
6	55	Femal
7	60	Femal
8	63	Femal
9	70	Femal
10	73	Femal
11	79	Femal
12	73	Femal
13	63	Male
14	64	Male
15	64	Male
16	66	Male
17	68	Male
18	69	Male
19	70	Male
20	70	Male
