# Supplementary material for: A Multiplex Protein Panel Applied to Cerebrospinal Fluid Reveals Three New Biomarker Candidates in ALS but None in Neuropathic Pain Patients
Source: PLoS One. 2016 Feb 25;11(2):e0149821. doi: 10.1371/journal.pone.0149821 (PMC4767403; doi:10.1371/journal.pone.0149821)
Supplement: S2 Table — (PDF) [file pone.0149821.s006.pdf]

S2 table. Antibodies and DNA oligonucleotides.

Antibody	R&D #	Arm 1	5'Mod	Sequence	Arm 2	5'Mod	Sequence	3'Mod	primer 1	Sequence	Primer 2	Sequence
Artemin	AF2589	Arm1-1	Thiol	AAAAACGATTCGAGAACGTGACTGCCATGGCTATTATGATGTCTGAGGCCCTATCCCTG	Arm2-1	Phos	AGTCTATATGCCCGATGCTGAATTAGTATCGATCGATACCGGACCAGGTTTCGCAAAAA	Thiol	SP_A1	GCTATTATGATGTCTGAGGC	SP_B1	GATACTAATTCAGCATCGGG
beta-NGF	AF-256	Arm1-2	Thiol	AAAAACGATTCGAGAACGTGACTGCCATGTACCTCTATTGATACGTGGGTATCCCTGAG	Arm2-2	Phos	TCTATATGATGACCTCACATTGACCATAAGGATCGATACCGGACCAGGTTTCGCAAAAA	Thiol	SP_A2	TACCTCTATTGATACGTGGG	SP_B2	CTTATGGTCAATGTGAGGTC
CathepsinB	AF953	Arm1-3	Thiol	AAAAACGATTCGAGAACGTGACTGCCATGAATAGAATCCCTACGCCTAGCCCTGAGTCT	Arm2-3	Phos	ATATGATGACGAGCTGAGTGAATCTTTCTCGATCGATACCGGACCAGGTTTCGCAAAAA	Thiol	SP_A3	AATAGAATCCCTACGCCTAG	SP_B3	GAGAAAGATTCACTCAGCTC
CathepsinS	AF1183	Arm1-4	Thiol	AAAAACGATTCGAGAACGTGACTGCCATGCTTTCAAGTACCTTAGCTCGCCTGAGTCTA	Arm2-4	Phos	TATGATGACTCCCGTTAGACTATCTGCTATGATCGATACCGGACCAGGTTTCGCAAAAA	Thiol	SP_A4	CTTTCAAGTACCTTAGCTCG	SP_B4	ATAGCAGATAGTCTAACGGG
CCL16	AF802	Arm1-5	Thiol	AAAAACGATTCGAGAACGTGACTGCCATGGCTCGTTATGTATGTAGACGCTGAGTCTAT	Arm2-5	Phos	ATGATGACTAAAGTCTATATCAGTAGCGGGGATCGATACCGGACCAGGTTTCGCAAAAA	Thiol	SP_A5	GCTCGTTATGTATGTAGACG	SP_B5	CCCGCTACTGATATAGACTT
CCL2	AF-279	Arm1-6	Thiol	AAAAACGATTCGAGAACGTGACTGCCATGATATCAGCTAATAGGGTCGGTGAGTCTATA	Arm2-6	Phos	TGATGACTATCCGGGTAATGAATGATAACCGATCGATACCGGACCAGGTTTCGCAAAAA	Thiol	SP_A6	ATATCAGCTAATAGGGTCGG	SP_B6	GGTTATCATTCATTACCCGG
CCL4	AF-271	Arm1-7	Thiol	AAAAACGATTCGAGAACGTGACTGCCATGTAGTCAGGTTGGATGTCTACATCCCTGAGT	Arm2-7	Phos	CTATATGATGTAGATACCGATACAGTGCTCGATCGATACCGGACCAGGTTTCGCAAAAA	Thiol	SP_A7	TAGTCAGGTTGGATGTCTAC	SP_B7	GAGCACTGTATCGGTATCTA
CCL5	AF-278	Arm1-8	Thiol	AAAAACGATTCGAGAACGTGACTGCCATGCCTATCCGTCTACGTGAATAGAGTCTATAT	Arm2-8	Phos	GATGACTATCAGATGTAGAGCATATACCGGGATCGATACCGGACCAGGTTTCGCAAAAA	Thiol	SP_A8	CCTATCCGTCTACGTGAATA	SP_B8	CCGGTATATGCTCTACATCT
CD40Ligand	AF617	Arm1-9	Thiol	AAAAACGATTCGAGAACGTGACTGCCATGAAGGTAATCTACACTACGGGGTCTATATGA	Arm2-9	Phos	TGACTATCGTCACATACGCTACAGTTACAGGATCGATACCGGACCAGGTTTCGCAAAAA	Thiol	SP_A9	AAGGTAATCTACACTACGGG	SP_B9	CTGTAACTGTAGCGTATGTG
Coagulation Factor III	AF2339	Arm1-10	Thiol	AAAAACGATTCGAGAACGTGACTGCCATGCAATCATATCTAACCGGCTGCTATATGATG	Arm2-10	Phos	ACTATCGTACGCCCGTATGGTAACATTATCGATCGATACCGGACCAGGTTTCGCAAAAA	Thiol	SP_A10	CAATCATATCTAACCGGCTG	SP_B10	GATAATGTTACCATACGGGC
CXCL5	AF254	Arm1-11	Thiol	AAAAACGATTCGAGAACGTGACTGCCATGACTATCCGTCACTCAAGTAGTATATGATGA	Arm2-11	Phos	CTATCGTACCCAGTAAGTATAACGAGCTGCGATCGATACCGGACCAGGTTTCGCAAAAA	Thiol	SP_A11	ACTATCCGTCACTCAAGTAG	SP_B11	GCAGCTCGTTATACTTACTG
CystatinB	AF1408	Arm1-12	Thiol	AAAAACGATTCGAGAACGTGACTGCCATGCCGATACTTAAAGCGTAGTGATATGATGAC	Arm2-12	Phos	TATCGTACCCCACCGACGCTAATAGTTAAGGATCGATACCGGACCAGGTTTCGCAAAAA	Thiol	SP_A12	CCGATACTTAAAGCGTAGTG	SP_B12	CTTAACTATTAGCGTCGGTG
CystatinC	AF1196	Arm1-13	Thiol	AAAAACGATTCGAGAACGTGACTGCCATGGCTTACTATTCATACTGCCGTATGATGACT	Arm2-13	Phos	ATCGTACCCTGCGACGTATAGATTCGTATGGATCGATACCGGACCAGGTTTCGCAAAAA	Thiol	SP_A13	GCTTACTATTCATACTGCCG	SP_B13	CATACGAATCTATACGTCGC
EGF	AF236	Arm1-14	Thiol	AAAAACGATTCGAGAACGTGACTGCCATGCCTATCGCTCTATATCTGGGATGATGACTA	Arm2-14	Phos	TCGTACCCTGTAGCACTGGACATAGATGAGGATCGATACCGGACCAGGTTTCGCAAAAA	Thiol	SP_A14	CCTATCGCTCTATATCTGGG	SP_B14	CTCATCTATGTCCAGTGCTA
E-Selectin	AF724	Arm1-15	Thiol	AAAAACGATTCGAGAACGTGACTGCCATGCGCTCTTATACCACTGTAGAGATGACTATC	Arm2-15	Phos	GTACCCTGCACGGTACTCAGTCATAGTGATGATCGATACCGGACCAGGTTTCGCAAAAA	Thiol	SP_A15	CGCTCTTATACCACTGTAGA	SP_B15	ATCACTATGACTGAGTACCG
TNFRSF6	AF326	Arm1-16	Thiol	AAAAACGATTCGAGAACGTGACTGCCATGTAGACTGATTGCTACTACGGATGACTATCG	Arm2-16	Phos	TACCCTGCAAATCCTCGATGGTAATGTAGGGATCGATACCGGACCAGGTTTCGCAAAAA	Thiol	SP_A16	TAGACTGATTGCTACTACGG	SP_B16	CCTACATTACCATCGAGGAT
Follistatin	AF669	Arm1-17	Thiol	AAAAACGATTCGAGAACGTGACTGCCATGCGCGTATCTCTCTCTAGTAGTGACTATCGT	Arm2-17	Phos	ACCCTGCAACGAGATGACGTGACATATTCGGATCGATACCGGACCAGGTTTCGCAAAAA	Thiol	SP_A17	CGCGTATCTCTCTCTAGTAG	SP_B17	CGAATATGTCACGTCATCTC
GDF-15	AF957	Arm1-18	Thiol	AAAAACGATTCGAGAACGTGACTGCCATGGCAGTCTATTCCACTCAATGGACTATCGTA	Arm2-18	Phos	CCCTGCAACTCCCGCATTATTATACACGAGGATCGATACCGGACCAGGTTTCGCAAAAA	Thiol	SP_A18	GCAGTCTATTCCACTCAATG	SP_B18	CTCGTGTATAATAATGCGGG
Growth Hormone	AF1067	Arm1-19	Thiol	AAAAACGATTCGAGAACGTGACTGCCATGGTAACTGCTTCGTATCAAGGACTATCGTAC	Arm2-19	Phos	CCTGCAACTGGCCCGTTCTCTACAGTATATGATCGATACCGGACCAGGTTTCGCAAAAA	Thiol	SP_A19	GTAACTGCTTCGTATCAAGG	SP_B19	ATATACTGTAGAGAACGGGC
ICAM-1	AF720	Arm1-20	Thiol	AAAAACGATTCGAGAACGTGACTGCCATGCGTCATCATTACTCACAGTGCTATCGTACC	Arm2-20	Phos	CTGCAACTGTCAGACGACTATCGCATAATGGATCGATACCGGACCAGGTTTCGCAAAAA	Thiol	SP_A20	CGTCATCATTACTCACAGTG	SP_B20	CATTATGCGATAGTCGTCTG
IL-1 alpha	AF-200	Arm1-21	Thiol	AAAAACGATTCGAGAACGTGACTGCCATGGCGTGTGATTCCTAGTAATGCGTACCCTGC	Arm2-21	Phos	AACTGTCGCAGCTCCTCGCTAATAAGAAACGATCGATACCGGACCAGGTTTCGCAAAAA	Thiol	SP_A21	GCGTGTGATTCCTAGTAATG	SP_B21	GTTTCTTATTAGCGAGGAGC
IL-10	AF-217	Arm1-22	Thiol	AAAAACGATTCGAGAACGTGACTGCCATGCCAGCTCGTACTATCGAATAATCGTACCCT	Arm2-22	Phos	GCAACTGTCGCAGGTGTATAGAGTTCTGCTGATCGATACCGGACCAGGTTTCGCAAAAA	Thiol	SP_A22	CCAGCTCGTACTATCGAATA	SP_B22	AGCAGAACTCTATACACCTG
IL-17	AF317	Arm1-23	Thiol	AAAAACGATTCGAGAACGTGACTGCCATGCCGCACTCTCGTAATATAGAGTACCCTGCA	Arm2-23	Phos	ACTGTCGCATCCGGTTATCCAAGTGTACTAGATCGATACCGGACCAGGTTTCGCAAAAA	Thiol	SP_A23	CCGCACTCTCGTAATATAGA	SP_B23	TAGTACACTTGGATAACCGG
IL-6	AF-206	Arm1-24	Thiol	AAAAACGATTCGAGAACGTGACTGCCATGTATTCTAGTTCTGGACACGGTACCCTGCAA	Arm2-24	Phos	CTGTCGCATCCATACTCTTGAAGCGATGTCGATCGATACCGGACCAGGTTTCGCAAAAA	Thiol	SP_A24	TATTCTAGTTCTGGACACGG	SP_B24	GACATCGCTTCAAGAGTATG
IL-7	AF207	Arm1-25	Thiol	AAAAACGATTCGAGAACGTGACTGCCATGGTAACACTTGAGATGCAGTCACCCTGCAAC	Arm2-25	Phos	TGTCGCATCTCCTGTCCTACAACGAATAGAGATCGATACCGGACCAGGTTTCGCAAAAA	Thiol	SP_A25	GTAACACTTGAGATGCAGTC	SP_B25	TCTATTCGTTGTAGGACAGG
IL-8	AF-208	Arm1-26	Thiol	AAAAACGATTCGAGAACGTGACTGCCATGGTACTTCTGAAACGTGATGGCCCTGCAACT	Arm2-26	Phos	GTCGCATCTGCACGGTATTTAATCGGATGGGATCGATACCGGACCAGGTTTCGCAAAAA	Thiol	SP_A26	GTACTTCTGAAACGTGATGG	SP_B26	CCATCCGATTAAATACCGTG
PSA	AF1344	Arm1-27	Thiol	AAAAACGATTCGAGAACGTGACTGCCATGCCATAGTATCCTGTAAGCGTCCTGCAACTG	Arm2-27	Phos	TCGCATCTGTCTACATAGACGATAGGGACGGATCGATACCGGACCAGGTTTCGCAAAAA	Thiol	SP_A27	CCATAGTATCCTGTAAGCGT	SP_B27	CGTCCCTATCGTCTATGTAG
KLK6	AF2008	Arm1-28	Thiol	AAAAACGATTCGAGAACGTGACTGCCATGACTGCTCAATCCTAGATACGCTGCAACTGT	Arm2-28	Phos	CGCATCTGTAACACTCACGACATATAGGTCGATCGATACCGGACCAGGTTTCGCAAAAA	Thiol	SP_A28	ACTGCTCAATCCTAGATACG	SP_B28	GACCTATATGTCGTGAGTGT
Mouse IgG	AF007	Arm1-29	Thiol	AAAAACGATTCGAGAACGTGACTGCCATGAGAGCATATCCTACTCGTTGTGCAACTGTC	Arm2-29	Phos	GCATCTGTAGCCTATCCGTACATATCGAGGGATCGATACCGGACCAGGTTTCGCAAAAA	Thiol	SP_A29	AGAGCATATCCTACTCGTTG	SP_B29	CCTCGATATGTACGGATAGG
p53	AF1355	Arm1-30	Thiol	AAAAACGATTCGAGAACGTGACTGCCATGAATAGACATCAGTAGCTCCGGCAACTGTCG	Arm2-30	Phos	CATCTGTAGCGCCTAGTCACCAATATACGAGATCGATACCGGACCAGGTTTCGCAAAAA	Thiol	SP_A30	AATAGACATCAGTAGCTCCG	SP_B30	TCGTATATTGGTGACTAGGC
P-Selectin	AF137	Arm1-31	Thiol	AAAAACGATTCGAGAACGTGACTGCCATGGTGACAGATTCTATGTGTGCCAACTGTCGC	Arm2-31	Phos	ATCTGTAGCACTTCATATAGTGAGAGGCGTGATCGATACCGGACCAGGTTTCGCAAAAA	Thiol	SP_A31	GTGACAGATTCTATGTGTGC	SP_B31	ACGCCTCTCACTATATGAAG
TIMP-1	AF970	Arm1-32	Thiol	AAAAACGATTCGAGAACGTGACTGCCATGCCCGATATTACTCGAACTGAAACTGTCGCA	Arm2-32	Phos	TCTGTAGCAGATAGAGACTTGGACATAGCGGATCGATACCGGACCAGGTTTCGCAAAAA	Thiol	SP_A32	CCCGATATTACTCGAACTGA	SP_B32	CGCTATGTCCAAGTCTCTAT
TIMP-4	AF974	Arm1-33	Thiol	AAAAACGATTCGAGAACGTGACTGCCATGCGGTCCTCTGCATCTATAATACTGTCGCAT	Arm2-33	Phos	CTGTAGCAGTCTCAGCCGAGTATATTGACTGATCGATACCGGACCAGGTTTCGCAAAAA	Thiol	SP_A33	CGGTCCTCTGCATCTATAAT	SP_B33	AGTCAATATACTCGGCTGAG
TNF-alpha	AF210	Arm1-34	Thiol	AAAAACGATTCGAGAACGTGACTGCCATGCCGCTGCATCTACTATAAGATGTCGCATCT	Arm2-34	Phos	GTAGCAGTCTCATTTGCAGTAAGTCGGTAGGATCGATACCGGACCAGGTTTCGCAAAAA	Thiol	SP_A34	CCGCTGCATCTACTATAAGA	SP_B34	CTACCGACTTACTGCAAATG
CCL3	AF-270-NA	Arm1-35	Thiol	AAAAACGATTCGAGAACGTGACTGCCATGGCTCTAATGTTAAGTGCTCGGTCGCATCTG	Arm2-35	Phos	TAGCAGTCTGCACGAGTGCTAAGATTTGAGGATCGATACCGGACCAGGTTTCGCAAAAA	Thiol	SP_A35	GCTCTAATGTTAAGTGCTCG	SP_B35	CTCAAATCTTAGCACTCGTG
CX3CL1	AF365	Arm1-36	Thiol	AAAAACGATTCGAGAACGTGACTGCCATGAGAATACTCTACATCGAGCGTCGCATCTGT	Arm2-36	Phos	AGCAGTCTGCGCCCTATACGAACATTTGACGATCGATACCGGACCAGGTTTCGCAAAAA	Thiol	SP_A36	AGAATACTCTACATCGAGCG	SP_B36	GTCAAATGTTCGTATAGGGC
GDNF	AF-212-NA	Arm1-37	Thiol	AAAAACGATTCGAGAACGTGACTGCCATGGTCGCATATCGTTCTACCTACGCATCTGTA	Arm2-37	Phos	GCAGTCTGCCAGGCAGATACGATTAGTACGGATCGATACCGGACCAGGTTTCGCAAAAA	Thiol	SP_A37	GTCGCATATCGTTCTACCTA	SP_B37	CGTACTAATCGTATCTGCCT
IL1b	AF-201-NA	Arm1-38	Thiol	AAAAACGATTCGAGAACGTGACTGCCATGGGCGCATATCTGTCTATACTGCATCTGTAG	Arm2-38	Phos	CAGTCTGCCGCGACGATGCTTATAGACTCTGATCGATACCGGACCAGGTTTCGCAAAAA	Thiol	SP_A38	GGCGCATATCTGTCTATACT	SP_B38	AGAGTCTATAAGCATCGTCG
IL4	AF-204-NA	Arm1-39	Thiol	AAAAACGATTCGAGAACGTGACTGCCATGACTCGTATATCATAAGCCCGCATCTGTAGC	Arm2-39	Phos	AGTCTGCCGCCAACGGATAGTTAGTCAGGTGATCGATACCGGACCAGGTTTCGCAAAAA	Thiol	SP_A39	ACTCGTATATCATAAGCCCG	SP_B39	ACCTGACTAACTATCCGTTG
KLK5	AF1108	Arm1-40	Thiol	AAAAACGATTCGAGAACGTGACTGCCATGCAACTAAGTGCTACCGTCTAATCTGTAGCA	Arm2-40	Phos	GTCTGCCGCGGACTGCGTATCATATAGGCTGATCGATACCGGACCAGGTTTCGCAAAAA	Thiol	SP_A40	CAACTAAGTGCTACCGTCTA	SP_B40	AGCCTATATGATACGCAGTC
KLK14	AF2626	Arm1-41	Thiol	AAAAACGATTCGAGAACGTGACTGCCATGCTGATGCTTAACTCGTATGCCTGTAGCAGT	Arm2-41	Phos	CTGCCGCGCTGATACTACGACATCTAGGGCGATCGATACCGGACCAGGTTTCGCAAAAA	Thiol	SP_A41	CTGATGCTTAACTCGTATGC	SP_B41	GCCCTAGATGTCGTAGTATC
MMP2	AF902	Arm1-42	Thiol	AAAAACGATTCGAGAACGTGACTGCCATGCAAGCATCTCTACTAGCAGTTGTAGCAGTC	Arm2-42	Phos	TGCCGCGCTTCATACATCTGAAGTAAGCGCGATCGATACCGGACCAGGTTTCGCAAAAA	Thiol	SP_A42	CAAGCATCTCTACTAGCAGT	SP_B42	GCGCTTACTTCAGATGTATG
Nt3	AF-267-NA	Arm1-43	Thiol	AAAAACGATTCGAGAACGTGACTGCCATGCTCCTAGCTTATGACAGCATTAGCAGTCTG	Arm2-43	Phos	CCGCGCTTTCACTATCTAACGAGCTTGGTCGATCGATACCGGACCAGGTTTCGCAAAAA	Thiol	SP_A43	CTCCTAGCTTATGACAGCAT	SP_B43	GACCAAGCTCGTTAGATAGT
Nt4	AF-268-NA	Arm1-44	Thiol	AAAAACGATTCGAGAACGTGACTGCCATGAATCTATCTACAAGCCGGTCAGCAGTCTGC	Arm2-44	Phos	CGCGCTTTCGCACATCGTTTAACACTGGAGGATCGATACCGGACCAGGTTTCGCAAAAA	Thiol	SP_A44	AATCTATCTACAAGCCGGTC	SP_B44	CTCCAGTGTTAAACGATGTG
Sortilin	AF3154	Arm1-45	Thiol	AAAAACGATTCGAGAACGTGACTGCCATGGTTGTATAGTATTCGCGCAGGCAGTCTGCC	Arm2-45	Phos	GCGCTTTCGCCCAAGTAGTACCAACTGTGAGATCGATACCGGACCAGGTTTCGCAAAAA	Thiol	SP_A45	GTTGTATAGTATTCGCGCAG	SP_B45	TCACAGTTGGTACTACTTGG
VEGF	AF-293-NA	Arm1-46	Thiol	AAAAACGATTCGAGAACGTGACTGCCATGTACAGCTATTGGAGACGATGCAGTCTGCCG	Arm2-46	Phos	CGCTTTCGCACCCGTGCATCAATAGTAGTAGATCGATACCGGACCAGGTTTCGCAAAAA	Thiol	SP_A46	TACAGCTATTGGAGACGATG	SP_B46	TACTACTATTGATGCACGGG
IL18	AF646	Arm1-47	Thiol	AAAAACGATTCGAGAACGTGACTGCCATGCGCATATCTACGTCTCTAGGAGTCTGCCGC	Arm2-47	Phos	GCTTTCGCAGGACTATGTATTAAGCGTGCGGATCGATACCGGACCAGGTTTCGCAAAAA	Thiol	SP_A47	CGCATATCTACGTCTCTAGG	SP_B47	CGCACGCTTAATACATAGTC
MMP9	AF911	Arm1-48	Thiol	AAAAACGATTCGAGAACGTGACTGCCATGATGTATCCGAAGTCGTAGTGGTCTGCCGCG	Arm2-48	Phos	CTTTCGCAGTGCTGGATGAGTACGTCTATTGATCGATACCGGACCAGGTTTCGCAAAAA	Thiol	SP_A48	ATGTATCCGAAGTCGTAGTG	SP_B48	AATAGACGTACTCATCCAGC
		universal primer 1		CGATTCGAGAACGTGACTGC								
		universal primer 2		GCGAAACCTGGTCCGGTATC								
		connecting oligo		GGAUAGGGACUCAGAUAUACUACUGAUAGCAUGGGACGUUGACAGCGUAGACAUCGUCAGACGGCGCGAAAGCGUCAUA								
